# Toward Clinically Trustworthy Deep Learning in Cardiology: A Unified Mathematical Synthesis of Representation, Optimization, and Reliability

**DOI:** 10.3390/diagnostics16213622

**Published:** 2026-11-02

**Authors:** Waqas Ahmad, Boulbaba Ben Ammar, Khukola Ibrahim

**Affiliations:** 1 Department of Computer Science, Abdul Wali Khan University Mardan, Mardan 23200, Pakistan; khukolaibrahimawkum@gmail.com; 2 Department of Computer Science, College of Computer, Qassim University, Buraydah 52571, Saudi Arabia

**Keywords:** trustworthy artificial intelligence, deep learning, cardiology, mathematical modeling, explainable artificial intelligence (XAI), systematic review

## Abstract

**Background:** Deep learning (DL) has revolutionized cardiovascular medicine by allowing for automated analysis of electrocardiography, imaging of the heart, and multimodal clinical datasets. Although there have been impressive advancements in terms of predictive performance, the deployment of DL methods in a clinical setting remains limited due to lack of sufficient mathematical understanding around the concepts of uncertainty, robustness, fairness, explainability, and generalizability. **Methods:** In this work, a systematic review is conducted using PRISMA principles on 79 peer-reviewed articles published from 2017 until 2025, with a unifying mathematical perspective on the notion of trustworthy deep learning in cardiology. **Results:** We find that there is significant discrepancy between prediction accuracy and clinical reliability, with little emphasis placed on mathematical guarantees, reproducibility of evaluation procedures, calibration, or robustness verification. To address this challenge, we propose a conceptual mathematical framework which brings together the elements of representation learning, optimization, and trustworthiness into a unified audit of AI in cardiac care. We note that the presented indices are purely theoretical and are meant to provide guidance for future standardization, and as such do not represent actual clinical metrics. **Conclusions:** Although our paper does not propose any new predictive algorithms, we do contribute a strong theoretical basis for the evaluation and development of clinically reliable deep learning algorithms in cardiology.

## 1. Introduction

### 1.1. Clinical Motivation and the Need for Trustworthy Cardiac AI

Cardiovascular disease (CVD) is the most significant cause of death across the world. Despite considerable progress in diagnostic techniques and therapy, CVD continues to pose serious challenges on the clinical, societal, and economic levels, [1]. As stated in recent global health statistics, CVD represents almost a third of all deaths worldwide and affects hundreds of millions of people per year. Early diagnosis and risk assessment of CVD remains one of the most crucial issues in contemporary medicine [2]. A variety of medical technologies, including electrocardiography (ECG), echocardiography, cardiac magnetic resonance imaging (CMR), computed tomography (CT), wearable devices, and EHRs, have produced vast amounts of cardiovascular data [3]. Although there is great potential for precision cardiology thanks to such multimodal data, the resulting complexity often surpasses the capacities of traditional analytical approaches, creating the need for intelligent computing systems able to support clinical decision-making [4]. Cardiovascular diagnosis has always been difficult due to the fact that clinical decision making involves bringing together varied and often incomplete information in the form of physiological information, different medical image modalities, biomarkers from laboratory results, and patient information over time [5]. Diagnostic interpretation in cardiovascular medicine is further complicated by patient differences, medical imaging differences, noise, lack of clinical information, and changes in disease phenotype [6], especially for conditions such as atrial fibrillation, heart failure, coronary heart disease, cardiomyopathy, and myocardial infarction. Recently, there have been major breakthroughs in the field of artificial intelligence (AI) and deep learning (DL) which have completely transformed computational cardiology by means of hierarchical learning algorithms based on raw data [7]. Several kinds of neural architectures, including convolutional neural networks (CNNs), recurrent neural networks (RNNs), LSTM networks, vision transformers (ViTs), graph neural networks (GNNs), and multimodal learning frameworks have achieved remarkable success in a number of cardiovascular applications [8], including the detection of arrhythmias, diagnosis of myocardial infarctions, segmentation of cardiac images, prediction of heart failure, coronary artery disease evaluation, and patient risk stratification [9].

However, predictive accuracy alone is not sufficient to ensure safe application of these models in the clinic [10]. Deep learning models designed to aid in cardiovascular decision-making, require reliable estimates of prediction uncertainty, model interpretability, fairness, robustness to distributional shift, robustness to adversarial attacks, and adherence to ethical, regulatory, and privacy concerns [11]. Even otherwise very accurate models can generate unacceptable clinical results due to poor calibration, systematic bias, or failure to generalize across different groups of patients [12]. Thus, a recent trend is to move beyond optimizing the accuracy of the model and towards building trustworthy artificial intelligence models that incorporate reliability, transparency, robustness, fairness, privacy preservation, and clinical accountability features [13].

### 1.2. Mathematical Foundations of Trustworthy Cardiac AI

With increasing awareness regarding the trustworthiness of artificial intelligence, there have been increased calls for robust mathematical formalism to support cardiovascular deep learning [14]. While terms such as uncertainty quantification, explainability, fairness, robustness, privacy preservation, and model calibration have gained attention in the literature, they are mostly addressed as separate components of algorithms, without being interconnected as mathematical principles that can underlie trustworthy clinical decision-making. Safe clinical use of machine learning models requires theoretical formalism that describes the principles of representation learning, optimization, generalization, propagation of uncertainty, and probabilistic inference [15]. Lack of such formalism makes the objective assessment of model reliability, comparison of alternative methods, and reproducibility impossible [16].

**Figure 1:**
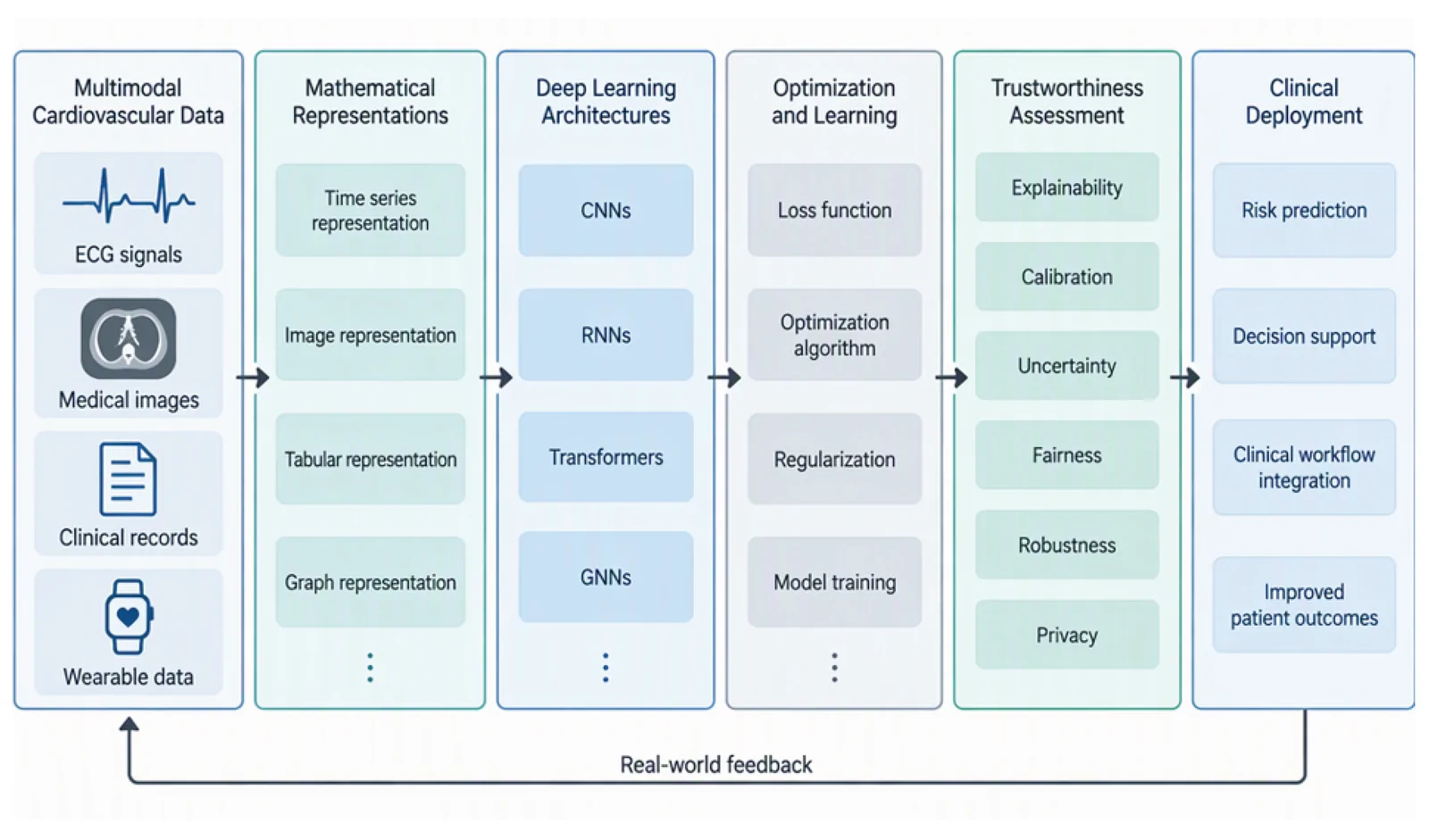
Overview of the end-to-end ecosystem linking multimodal cardiovascular data, mathematical representations, deep learning architectures, optimization theory, trustworthiness constraints, and clinical deployment.

While substantial progress has been made in the field of trustworthy AI, its mathematical underpinnings are still scattered between the different areas of explainability, uncertainty quantification, fairness-aware machine learning, adversarial machine learning, federated learning, and privacy-preserving computing [17]. Previous review articles have mostly concentrated either on methodological advancements or on the applications of AI in clinics, with much less effort being made to address the mathematical interrelationships among them [18]. Table 1 summarizes this gap relative to recent related reviews. It can be seen that the mathematical connections between optimization, statistics, uncertainty modeling, fairness considerations, robustness requirements, and clinic reliability have not been well articulated [19]. Figure 1 summarizes this end-to-end pipeline, linking multimodal cardiovascular data, mathematical representation, architecture choice, optimization, and trustworthiness constraints to clinical deployment.

As a means of filling this void, this paper conducts a systematic review following PRISMA guidelines in order to develop a unified theoretical framework on the mathematical aspects of trustworthy deep learning in cardiology [22]. Our analysis of 79 scientific papers published from 2017 to 2025 helps to uncover patterns of methodology, theoretical difficulties, and future research directions related to representation learning, optimization, explainability, uncertainty quantification, fairness, robustness, privacy-preserving learning, and statistical generalization; in other words, this review aims to provide a basis for the future use of predictive algorithms in clinical practice.

The primary goal of this review is to foster trust in cardiac AI systems by formulating an overarching theory from various mathematical ideas which will be helpful in building AI that is transparent, reproducible, regulation-ready, and safe for patients. This synthesis lays down the groundwork for further study in areas such as explainability in machine learning, clinical decision-making with awareness of uncertainty, optimization with constraints of fairness, robust validation of models, and privacy preservation in artificial intelligence [23].

### 1.3. Motivation and Contribution to the Literature

The current review provides an answer to the lack of integration in the field of mathematical fundamentals of trustworthy deep learning algorithms for cardiovascular purposes. Instead of considering only prediction accuracy or particular methods, it brings together representation learning, optimization, and different aspects of trustworthiness in order to analyze their relevance to clinical application. With the help of 79 articles synthesized into one mathematical picture, this review tries to fill the gap of existing methodologies in this area.

### 1.4. Summary of Major Contributions

The principal contributions of this systematic review are summarized as follows:

- **Comprehensive and systematic synthesis of evidence:** This review systematically synthesizes recent advances in trustworthy deep learning for cardiovascular medicine through a PRISMA-guided analysis of 79 primary studies published between 2017 and 2025, providing a comprehensive overview of the current research landscape.
- **Unified mathematical synthesis:** Rather than reviewing individual algorithms in isolation, this work organizes the mathematical principles underlying representation learning, optimization, uncertainty quantification, explainability, fairness, robustness, privacy-preserving learning, and statistical generalization into a unified conceptual mathematical perspective.
- **Critical evaluation of trustworthiness:** The reviewed literature is critically examined beyond conventional predictive performance by emphasizing clinically relevant dimensions such as calibration, uncertainty estimation, algorithmic fairness, robustness, interpretability, reproducibility, and clinical reliability.
- **Identification of research gaps:** This review identifies important theoretical, methodological, and translational challenges that continue to limit the safe deployment of deep learning models in real-world cardiovascular practice, while highlighting opportunities for future methodological development.
- **Conceptual framework for future research:** A structured conceptual mathematical framework is presented in order to connect the diverse components of trustworthy cardiac AI and support future research toward standardized evaluation and clinically reliable artificial intelligence systems.
- **Clinical and interdisciplinary perspective:** By integrating concepts from artificial intelligence, mathematics, statistics, and cardiovascular medicine, this review establishes a common theoretical foundation intended to facilitate interdisciplinary collaboration and accelerate the development of transparent, robust, and clinically trustworthy cardiac AI.
- **Novel mathematical auditing constructs (TDI and RD):** In addition to descriptive meta-synthesis, the current review proposes two novel mathematical measures: the Representation Deficit (RD), which offers a kernel-based framework for measuring the degree of manifold discrepancy in cardiovascular representation learning, and the Trustworthiness Deficit Index (TDI), which converts multi-dimensional trustworthiness (calibration, fairness, robustness, explainability) into an auditable scalar metric.
- **Mathematically-driven narrative synthesis methodology:** Finally, this review provides a narrative synthesis methodology that relates architectures (CNN, RNN, transformer, GNN) to particular trust deficit issues and proposes the RD and TDI framework for their future quantification.

### 1.5. Paper Organization

The rest of this paper is structured as follows: Section 2 covers the methodological aspects of the systematic review, namely, the literature search process, criteria for selecting studies, and quality assessment; Section 3 presents an overview of the mathematical underpinnings of modern deep learning architectures in the context of cardiology; Section 4 critically analyzes the mathematical basis of explainability, uncertainty quantification, fairness, robustness, and privacy-preserving learning; the critical synthesis and results of this investigation are provided in Section 5; in Section 6, we discuss the results and their implications; finally, the underlying mathematical framework resulting from the literature review is presented in Section 7. Section 8 concludes the paper.

## 2. Research Methodology

### 2.1. Study Design

This review was performed as a PRISMA systematic review [24] of the mathematical framework of trustworthy deep learning in cardiovascular medicine. A pre-defined review protocol was set up before starting the literature search in order to enhance methodological transparency, reproducibility, and consistency during the process of literature screening, methodology quality assessment, and evidence synthesis.

While traditional narrative reviews mainly summarize the existing literature, in this review we follow the approach of mathematically-driven evidence synthesis, i.e., systematic extraction and analysis of theoretical formulations relevant to representation learning, optimization, uncertainty quantification, explainability, fairness, robustness, privacy-preserving learning, and statistical generalizability. The purpose of this review is to determine the mathematical principles behind the creation, validation, and application of trustworthy deep learning models in cardiovascular disease contexts.

**Figure 2:**
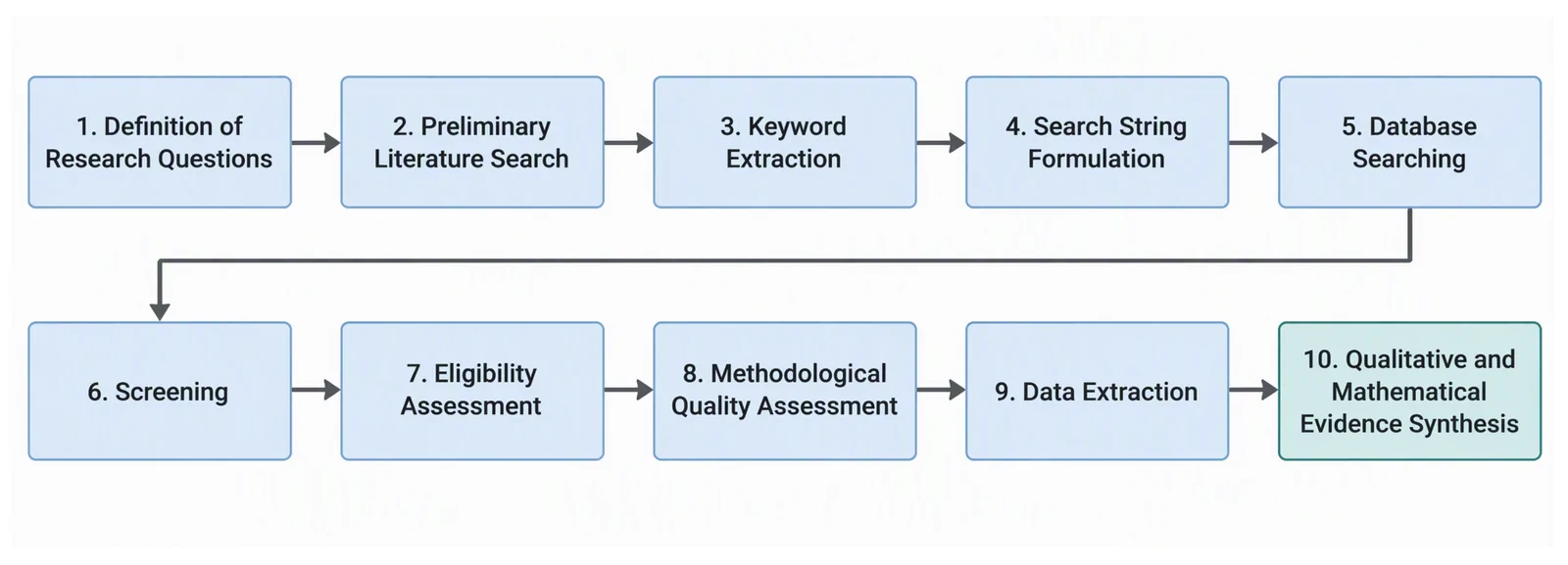
Workflow of the systematic review.

The systematic review was performed in five consecutive steps: (i) definition of research questions, (ii) systematic literature search, (iii) literature screening based on the pre-defined inclusion and exclusion criteria, (iv) assessment of the selected articles’ methodological quality, and (v) qualitative and mathematical synthesis of the selected articles. All steps of the article selection process are illustrated in Figure 2.

### 2.2. Research Questions

The main goal of this systematic review is to develop a thorough mathematical understanding of reliable deep learning concepts in cardiology through a critical synthesis of the available literature [25]. In order to accomplish this main goal, this systematic review is designed based on the following four theoretical research questions (RQs):

- **RQ1: Mathematical Representation of Cardiovascular Data**
  How are cardiovascular data mathematically represented and modeled within contemporary deep learning frameworks, and what theoretical formulations underpin these representations across different clinical modalities?
- **RQ2: Mathematical Foundations of Deep Learning Architectures**
  What mathematical principles govern the design, optimization, convergence, and generalization of deep learning architectures employed for cardiovascular diagnosis, prognosis, and clinical decision support?
- **RQ3: Mathematical Characterization of Trustworthy Artificial Intelligence**
  How are explainability, uncertainty quantification, fairness, robustness, privacy preservation, and model calibration mathematically formulated and evaluated in current cardiovascular deep learning research?
- **RQ4: Open Challenges and Future Research Directions**
  What theoretical limitations, methodological gaps, and unresolved mathematical challenges remain for developing clinically reliable, transparent, and trustworthy deep learning systems in cardiovascular medicine?

Collectively, these research questions provide the conceptual framework for organizing the evidence synthesized throughout this review and establish the logical structure for the subsequent sections of the manuscript.

### 2.3. Literature Search Strategy

**Figure 3:**
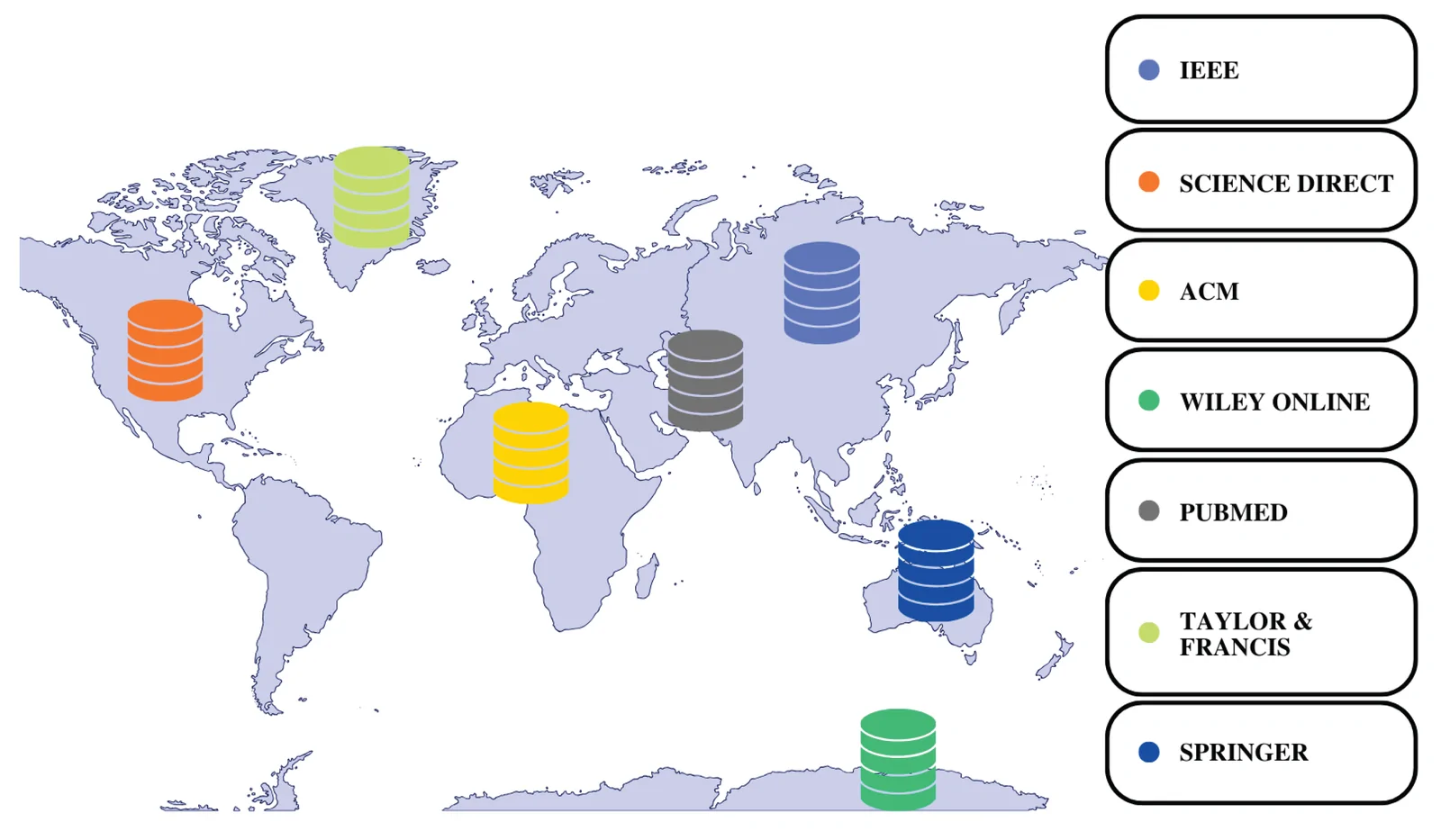
Databases searched.

A systematic search of the literature was devised to find peer-reviewed articles about the application of deep learning methods in cardiology. As per the PRISMA guidelines, the search strategy was formulated according to the population–concept–context (PCC) model and involved seven different scientific databases and publishing portals: IEEE Xplore, Springer, PubMed, ScienceDirect, ACM Digital Library, Wiley Online Library, and Taylor & Francis Online, as illustrated in Figure 3. The period of time chosen for the search process comprised all studies published from January 2017 up until June 2025, with the search process itself conducted within June–July 2025. This timeframe was chosen purposefully in order to be able to conduct a thorough audit of the studies found based on their mathematical content, meaning that every study should be analyzed based on its theoretical formalism, not its predictions. Thus, any studies published after June 2025 are omitted from the results.

To ensure a conceptually complete and replicable search strategy, four topic-specific thematic areas were considered for organizing the search process: (i) deep learning techniques, (ii) cardiovascular diseases and associated physiological signals, (iii) computational aspects, and (iv) prediction, diagnosis, and analysis tasks. Factors connected to the trustworthiness of the study, such as explainability, fairness, robustness, uncertainty quantification, and privacy, were listed as required keywords in the core search strings of all databases (Table 2). This strategy was chosen to ensure that the selected corpus would specifically cover theoretical and mathematical foundations of trustworthy AI, thereby narrowing the literature review to the relevant clinical reliability factors.

The general search strategy is represented mathematically as

(1) $$\begin{array}{cc} Q & =\left(\overset{N_{DL}}{\underset{i=1}{⋃}}C_{DL}^{\left(i\right)}\right)\cap \left(\overset{N_{Cardio}}{\underset{j=1}{⋃}}C_{Cardio}^{\left(j\right)}\right)\cap \left(\overset{N_{Method}}{\underset{k=1}{⋃}}M_{Method}^{\left(k\right)}\right)\cap \left(\overset{N_{Task}}{\underset{l=1}{⋃}}T_{Task}^{\left(l\right)}\right), \end{array}$$

where $C_{DL}$ refers to deep learning terminologies, $C_{Cardio}$ to cardiovascular disease terminologies, $M_{Method}$ to computational and methodology terminologies, and $T_{Task}$ to prediction, diagnosis, and analysis tasks [26]. Within each of the thematic domains, the alternatives were connected through the OR operator, whereas the domains were connected by means of the AND operator. Search strategies for individual databases were customized according to the needs of each platform while maintaining the basic search terms. The resulting search strings for each database are shown in Table 2.

Because of differences in the indexing and searching process for the different databases, the generic search strategy was accordingly adapted to the needs of each specific database while maintaining the overall idea behind the search strategy. The use of controlled vocabulary (for example, MeSH in PubMed) has been applied whenever possible, and Boolean operators have been used in the engineering-type databases. The returned articles were then exported for further steps in the review process.

### 2.4. Eligibility Criteria

Eligibility criteria were formulated to ensure an unbiased and standardized approach to selecting studies for review. The selection process included screening the titles, abstracts, and full articles. These criteria depended on the relevancy of each study with regard to deep learning in cardiovascular medicine.

**Inclusion Criteria**
  - Peer-reviewed journal articles and conference papers published between January 2017 and June 2025.
  - Original studies investigating deep learning in cardiovascular diagnosis, prognosis, risk prediction, medical image analysis, physiological signal analysis, or clinical decision support.
  - Studies reporting sufficient methodological information to assess the deep learning approach, including its architecture, training or optimization procedure, evaluation strategy, or clinical application.
  - Studies providing information relevant to at least one review objective, including model representation, optimization, validation, interpretability, fairness, robustness, uncertainty, privacy, or other aspects of clinical reliability, where reported.
  - Publications written in English.
  - Original research providing a quantitative evaluation or a substantive methodological contribution.

Research that examined deep learning in the fields of cardiovascular diagnosis, prognosis, risk prediction, physiological signal or medical image analysis, or decision support was considered eligible if there was enough methodological detail to describe the model and its validation. While reporting of the trustworthiness dimensions was not a requirement for eligibility, these dimensions were extracted whenever they were reported.

**Exclusion Criteria**
  - Editorials, letters, book chapters, dissertations, patents, abstracts, and other non-peer-reviewed publications.
  - Studies unrelated to cardiovascular medicine or that do not employ deep learning techniques.
  - Articles lacking sufficient methodological information to assess their relevance to the review objectives or for which the full text could not be obtained.
  - Duplicate records identified across the searched databases and platforms.
  - Non-English publications.

Terms related to trustworthiness were not necessary for inclusion; rather, the inclusion and handling of relevant dimensions were evaluated based on the whole text during data abstraction. Inclusion of studies was not limited based on whether they addressed explainability, fairness, robustness, uncertainty quantification, or privacy provided that the studies met all of the inclusion criteria. The inclusion criteria were uniformly applied when screening titles, abstracts, and full texts.

### 2.5. Study Selection Process

Articles were selected based on the PRISMA guidelines [26] following a multi-step selection process. Records retrieved from the databases were loaded in the reference manager to screen out duplicates automatically, while manual confirmation was done afterwards. The remaining records were then screened one-by-one by title, abstract, and full text based on the inclusion criteria provided in Section 2.4. Two reviewers performed the screening process independently at each step. Conflicts between the two reviewers were resolved through discussion; in case of unresolved conflicts, a third reviewer intervened.

The screening workflow is mathematically represented as

(2) $$\begin{array}{cc} S_{final} & =F_{Eligibility}(F_{Abstract}(\\ \; & \;F_{Title}\left(F_{Duplicate}\left(S_{retrieved}\right)\right))), \end{array}$$

where $S_{retrieved}$ is the set of all retrieved records, $F_{Duplicate}$ is the elimination of duplicates, $F_{Title}$ and $F_{Abstract}$ are title/abstract screening, and $F_{Eligibility}$ is full-text screening. Thus, the obtained set of studies $S_{final}$ consists of all studies included in the qualitative and mathematical syntheses.

**Figure 4:**
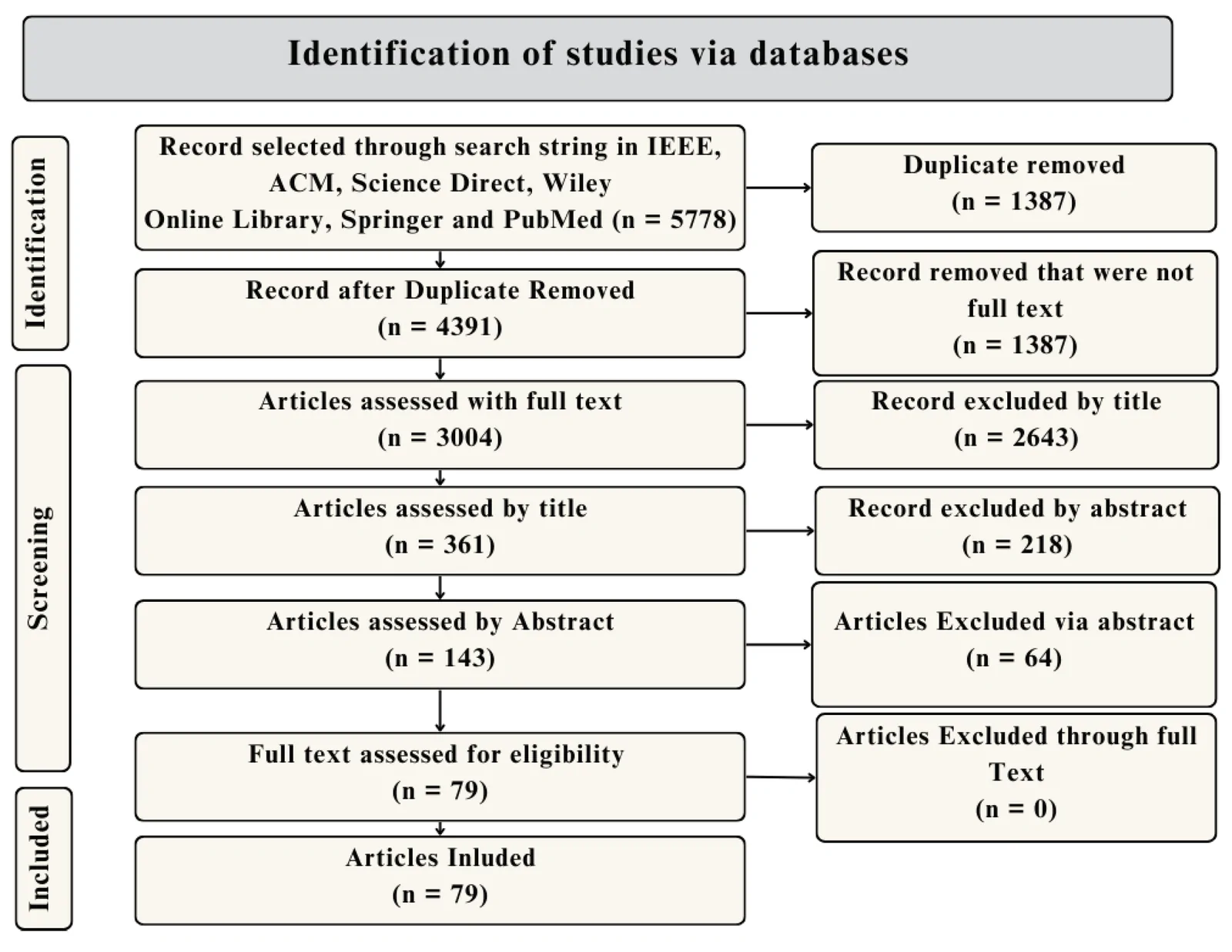
PRISMA flow diagram of study identification, screening, eligibility, and inclusion.

**Figure 5:**
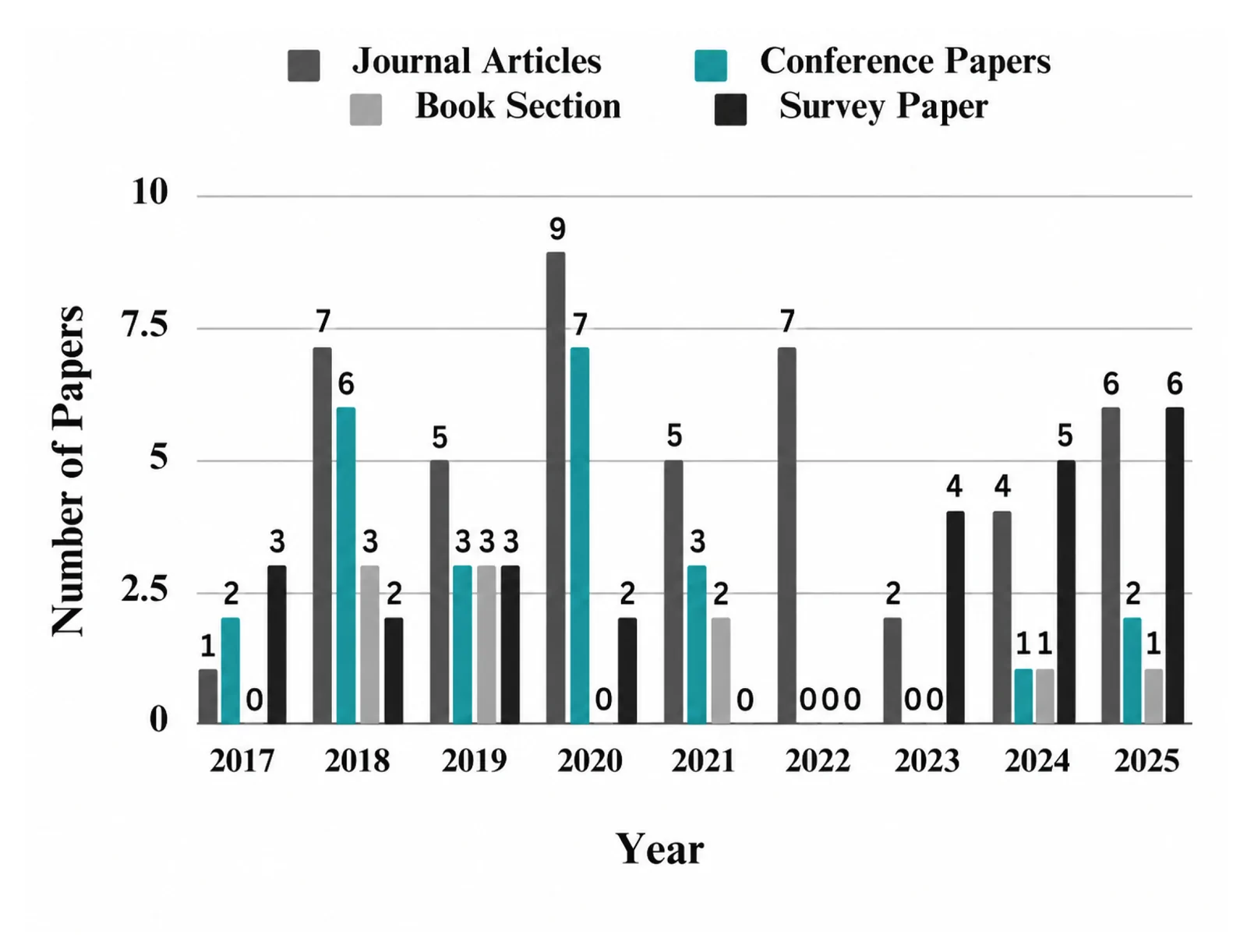
Included studies by year (2017–2025).

**Figure 6:**
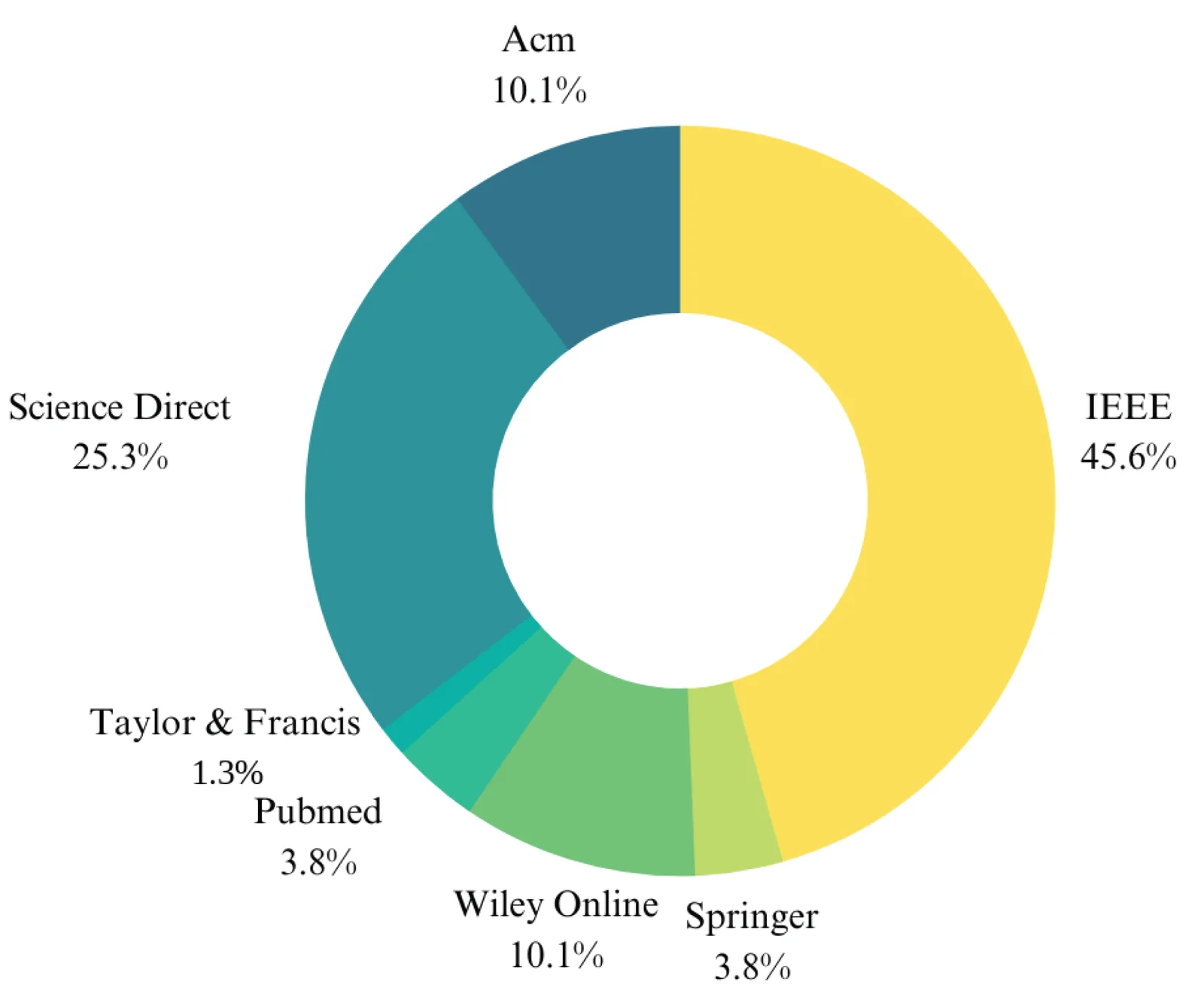
Distribution of databases by percentage.

The full screening process is depicted in the PRISMA flow chart (Figure 4), which shows the number of identified, screened, excluded, and included studies along with the major reasons for full-text exclusion. Following the search in seven databases, 5778 papers were initially identified. After eliminating 1387 duplicate papers using automated and manual methods, 4391 papers were left for further screening. Records without accessible full text were then removed, leaving 3004 papers for title screening. Of these 3004 papers, 2643 were excluded based on the title, leaving 361 papers for abstract-based screening. After abstract screening, 143 papers were left for full-text eligibility assessment. At the end of full-text (content) screening against the eligibility criteria in Section 2.4, 79 studies were left for the qualitative and mathematical syntheses. The numbers per database are given in Table 3. The yearly distribution of the selected articles is depicted in Figure 5. The proportional contribution of each database to the final corpus is shown in Figure 6.

### 2.6. Methodological Quality Assessment

The methodological quality of the studies used in the review was independently determined in order to test the validity, clarity, and rigorousness of the methods used in studies dealing with the use of deep learning methodologies. Since the purpose of this review is to determine the mathematics behind trustworthy artificial intelligence for cardiovascular care, an evaluation framework was designed [27]. It considers the following four methodological aspects:

1. Mathematical formulation and theoretical rigor.
2. Experimental design and reproducibility.
3. Evaluation of trustworthiness properties (e.g., explainability, uncertainty, fairness, robustness, and privacy).
4. Transparency of datasets, implementation details, and validation protocols.

Each dimension was rated on an ordinal scale from 0 to 1 with three levels: 0 means criterion not covered, 0.5 means partial coverage, and 1 means full coverage. We define the Methodological Quality Score (MQS) as follows:

(3) $$MQS=\sum_{i=1}^{4}w_{i}q_{i},$$

where $q_{i}$ is the rating of the *i*-th methodologicalcriterion and $w_{i}$ is the weight of the corresponding criterion [28]. Equal weights were defined ($w_{i}=1$ for all four criteria), resulting in possible MQS values ranging from 0 to 4. The MQS was not computed for the included studies; Equation (3) describes the proposed scoring procedure.

The methodology quality assessment framework was developed prior to the process and independently assessed by two reviewers. Any differences in assessment between the two reviewers were discussed until reaching a consensus. Unresolved differences were adjudicated by a third reviewer. No inter-rater agreement coefficient was computed. The proposed MQS framework is meant to guide the critical appraisal of the methodological strengths and weaknesses, and is not meant for use as a screening tool. Table 4 summarizes the relevance classification of the 79 articles included in the review with regard to the four research questions (RQ1–RQ4). The relevance classification and the total relevance score are different from the proposed MQS framework.

### 2.7. Data Extraction

The data extraction process was done by two independent reviewers using a predetermined data extraction form. The data extraction process involved collecting data such as bibliographic details, cardiovascular applications, modality and features of the dataset, deep learning models, mathematics used, optimization techniques, trustworthiness factors, and evaluation processes.

The following data were extracted for each of the eligible studies:

- Bibliographic information (authors, publication year, journal, and country).
- Cardiovascular application and clinical objective.
- Data modality (e.g., ECG, echocardiography, cardiac MRI, CT, electronic health records, or multimodal data).
- Dataset characteristics, including source, sample size, and availability.
- Deep learning architectures, mathematical formulations, optimization algorithms, loss functions, and training strategies.
- Trustworthiness components, including explainability, uncertainty quantification, fairness, robustness, privacy-preserving learning, and calibration.
- Performance metrics, validation strategies, and reported clinical outcomes.
- Mathematical models, theoretical analyses, and methodological contributions relevant to trustworthy deep learning.

The gathered data were classified into evidence tables and thematic categories for purposes of qualitative comparison and mathematical synthesis between the selected studies. The classification procedure allowed for an objective analysis of the methodology and theories presented in the reviewed literature.

### 2.8. Qualitative and Mathematical Evidence Synthesis

After data extraction, the studies were analyzed using an integrated qualitative and mathematical evidence synthesis approach. A quantitative meta-analysis was not conducted due to significant differences between datasets, applications in cardiovascular medicine, deep learning model architectures, methods for evaluating the models, and performance metrics.

A structured qualitative analysis was supplemented with a comparative mathematical analysis of the theory behind trustworthy deep learning.

The qualitative analysis first organized the studies into themes based on their cardiovascular application, data type, deep learning architecture, optimization technique, elements of trustworthiness, evaluation method, and clinical endpoints. This allowed us to detect research trends, methodological weaknesses, promising areas of study, and current gaps in the field.

The entire synthesis procedure may be formally defined as follows:

(4) $$K=\Phi \left(\overset{N}{\underset{i=1}{⋃}}E_{i}\right)$$

where $E_{i}$ is the structured evidence extracted from the *i*-th study, ${⋃}_{i=1}^{N}E_{i}$ is the complete evidence body, and Φ(·) is a thematic and mathematical synthesis operator that combines representation learning, optimization, uncertainty quantification, explainability, fairness, robustness, privacy-preserving learning, and statistical generalization into one coherent framework [22].

Having synthesized the evidence, we then systematically arranged it according to the established research questions (Section 2.2) in order to ensure consistency between the review objectives, evidence extraction, and mathematical treatment in the rest of the paper.

### 2.9. Risk of Bias and Methodological Limitations

Several steps were taken to avoid possible sources of bias and increase the reliability of the systematic review. First of all, a broad search of the literature was conducted in different scientific databases by means of specific search strategies, with clear inclusion and exclusion criteria defined prior to screening the studies. Moreover, studies were independently selected by two reviewers; any differences in opinion were sorted out through discussions, and if consensus could not be reached, through adjudication by a third reviewer. The methodological quality appraisal and data extraction were independently carried out by two researchers, and any differences were sorted out through discussion and consensus. However, some limitations of the systematic review should be highlighted. Language and publication bias could occur because of limiting the systematic review to English-language and peer-reviewed studies. Additionally, the rapid development of deep learning suggests that studies on this topic published subsequent to the literature search could have been missed. The large differences between methodological approaches across different cardiovascular applications, data modalities, deep learning architectures, evaluation protocols, and measured outcomes made it impossible to conduct a quantitative meta-analysis; hence, a qualitative and mathematical synthesis of the evidence was carried out instead. Nevertheless, the review was conducted according to a transparent and reproducible methodology that allowed us to perform a rigorous synthesis of the evidence. The process of study selection and data extraction was done independently by two reviewers, while any disagreement during study selection which could not be sorted out by discussion was referred to a third reviewer. Disagreements about the data extraction process were resolved through discussion. We did not calculate any form of inter-reviewer agreement statistic, such as Cohen’s Kappa.

The procedure for the audit of the conceptual approach to organizing and assessing the mathematical and trustworthiness qualities of deep learning studies is described in Algorithm 1. Full quantitative calculations and ranking of MQS scores for all included studies did not form part of the current review; this procedure is presented only as an example for future quantitative use.
**Algorithm 1** Mathematically Constrained Systematic Literature Review (MC-SLR) Audit Pipeline**Require:** 
Set of studies $S=\{S_{1},S_{2},...,S_{N}\}$**Ensure:** 
Methodological quality score MQS, representation score RS, trustworthiness score TS  1:**for** each study $S_{i}\in S$ **do**  2:      Extract data representation strategy: tensor, temporal, image, graph, multimodal  3:      Evaluate mathematical formulation: presence of equations, assumptions, constraints  4:      Assess optimization transparency: optimizer, learning rate, convergence analysis  5:      Evaluate trustworthiness: explainability, fairness, robustness, uncertainty  6:      Compute component scores:$$MQS_{i}=\alpha R_{i}+\beta O_{i}+\gamma T_{i}$$
where $R_{i}$, $O_{i}$, and $T_{i}$ represent representation, optimization, and trustworthiness scores.  7:**end for**  8:Rank studies according to $MQS_{i}$      **return** {MQS,RS,TS}

## 3. Theoretical Analysis of Deep Learning Architectures in Cardiology

### 3.1. Overview of Deep Learning Architectures in Cardiovascular Medicine

Deep learning is revolutionizing cardiovascular artificial intelligence in the sense that it allows for automatically acquiring hierarchical representations from intricate biomedical data [108]. In contrast to conventional machine learning paradigms that rely heavily on hand-crafted features, deep learning algorithms learn discriminative representations straight from the data, delivering state-of-the-art accuracy in ECG analysis, cardiac imaging, arrhythmia detection, heart failure prediction, coronary artery disease diagnostics, and risk stratification [109].

The wide variety of cardiovascular data has led to the development of customized neural architectures. Convolutional neural networks (CNNs) are adept at modeling spatial features of medical images and ECG signals, whereas LSTM and GRU networks model temporal correlations in physiological signals [110]. More recently, transformer architectures have shown advantages in modeling long-range interactions using self-attention, while graph neural networks (GNNs) naturally lend themselves to modeling anatomical and relational cardiac structures [111].

**Figure 7:**
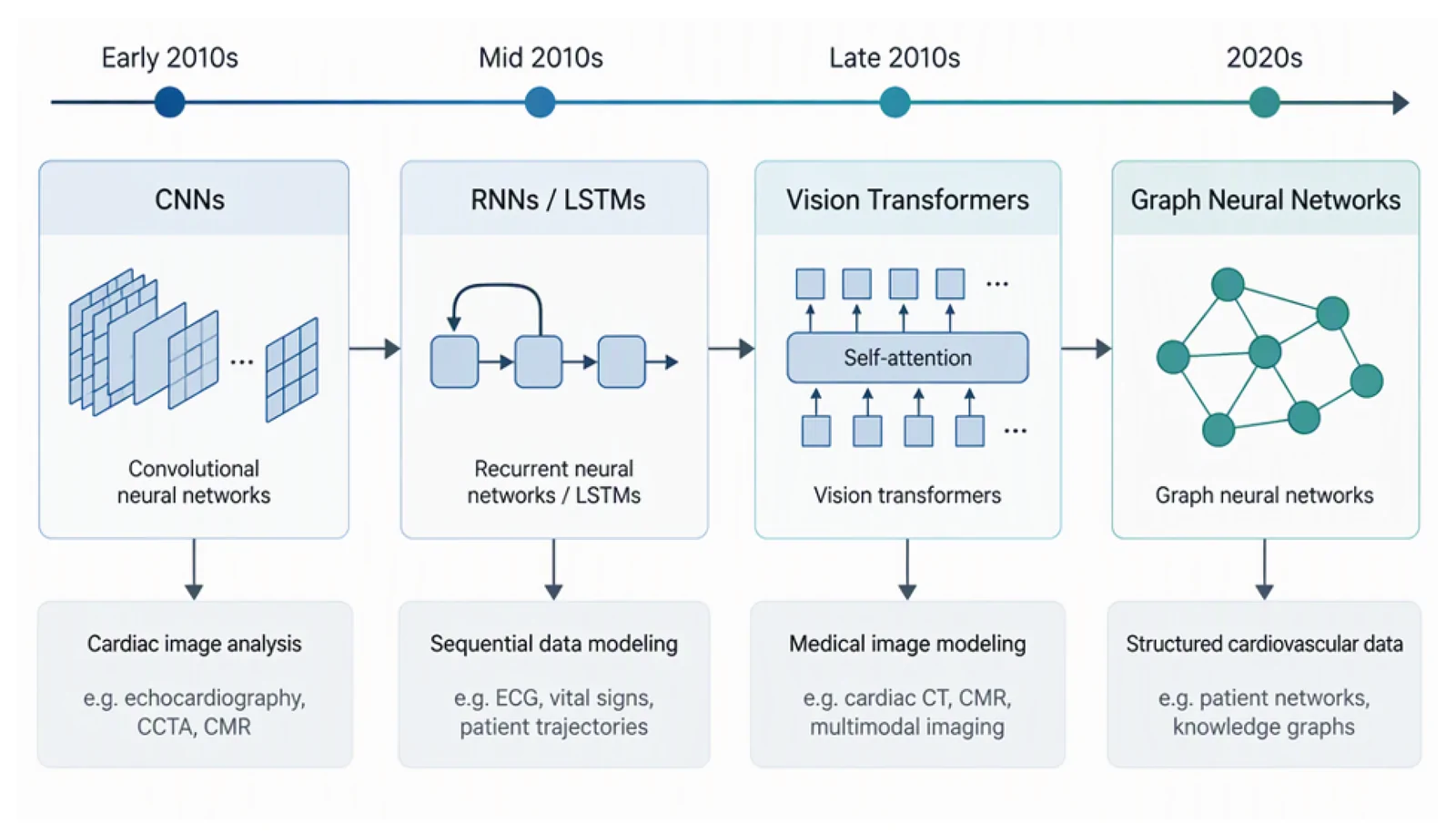
Chronologicalprogression from early convolutional neural networks to foundation models towards clinically trustworthy AI systems.

These architectures’ suitability is determined not just by their predictive accuracy but by their mathematical properties such as representational power, optimization dynamics, convergence, complexity, robustness, interpretability and generalization. The following subsections investigate the major deep learning architectures mathematically, focusing on the underlying theory that makes cardiovascular artificial intelligence trustworthy. Figure 7 traces this chronological progression from early CNN-based feature learning to current multimodal and foundation-model approaches towards future trustworthy cardiac AI.

### 3.2. Mathematical Representation of Cardiovascular Data

Mathematical modeling of cardiovascular data serves as the foundation of feature extraction, optimization, and inference in deep learning applications [112]. In contrast to traditional fixed-dimensional feature vectors, cardiovascular data consist of varying modalities such as time series of physiological measurements, medical images, volume series, graph-based anatomical data, and multimodal clinical information [113]. Choosing a suitable mathematical form is a critical step that preserves clinically meaningful information and allows for effective machine learning.

Various types of cardiovascular data have different mathematical representations. For example, electrocardiographic (ECG) recordings can be mathematically modeled as multivariate time series, while echocardiography and cardiac MRI are modeled as high-order tensors that account for spatial, temporal, and intensity information [114]. Anatomical structures and vascular networks can be presented by weighted graphs. Thus, there is a need to design dedicated deep learning architectures for specific data geometries.

When creating a uniform computational model, different cardiovascular data types are mathematically represented using tensor algebra, which preserves multidimensional relations and allows for feature extraction, multimodal data fusion, and hierarchical representation learning [115]. Thus, cardiovascular data can be expressed in terms of an *N*-dimensional tensor:

(5) $$X\in R^{I_{1}\times I_{2}\times \cdots \times I_{N}}$$

where $I_{n}$ can represent a clinical feature dimension such as spatial coordinates, temporal coordinates, channels for imaging data, physiological measures, or personal traits [116].

To facilitate computational efficiency and optimization, higher-order cardiovascular tensors are commonly converted to matrices using mode-*n* matricization (unfolding) [117]:

(6) $$X_{\left(n\right)}={\mathrm{Unfold}}_{n}\left(X\right)$$

with $X_{\left(n\right)}$ representing the mode-*n* unfolding of the tensor X. This retains multidimensionality while using tensor unfolding to perform optimization and neural network computations, which are traditionally carried out in matrix format [118].

In multi-lead electrocardiography, the obtained signal is represented by

(7) $$X\left(t\right)={\left[x_{1}\left(t\right),x_{2}\left(t\right),\ldots ,x_{L}\left(t\right)\right]}^{T},$$

where *L* is the number of ECG leads and $x_{i}\left(t\right)$ is the electrical potential obtained from the *i*-th lead at time *t* [119].

To reduce redundancy between ECG leads during the feature transformation process, the transformation matrix is encouraged toward the Stiefel manifold by the penalty

(8) $$L_{ortho}\left(W\right)=\frac{1}{2}{∥W^{T}W-I∥}_{F}^{2}.$$

This orthogonality constraint reduces feature redundancy, improves numerical stability during optimization, and promotes the learning of physiologically distinct latent representations across ECG leads [120].

### 3.3. Convolutional Neural Networks for Cardiovascular Representation Learning

Convolutional neural networks (CNNs) have been one of the most popular deep learning frameworks for the development of cardiovascular artificial intelligence thanks to their ability to learning hierarchical representation directly from physiological signals and medical images. By leveraging local correlations through weight sharing and sparse connections, CNNs have yielded impressive results in electrocardiogram (ECG) signal analysis, arrhythmia detection, myocardial infarction diagnosis, cardiac image segmentation, and anatomical structure detection. The translation-invariant nature of CNNs makes them suitable for the detection of clinically meaningful morphological features such as the QRS complex, ST-segment abnormality, ventricular boundaries, and myocardial tissue [121].

From a mathematical point of view, convolution is a localized linear transformation of the input tensor to kernel space. Denoting the input tensor by X and convolution kernel by K [122], the resultant feature map is given by

(9) $$Y\left(i,j\right)=\sum_{m}\sum_{n}K\left(m,n\right)X\left(i-m,j-n\right),$$

where successive convolutional layers successively map the low-level physiological patterns to high-level and discriminative cardiovascular patterns.

To preserve dominant structural information while reducing the spatial dimensionality, the process of convolution is usually followed by pooling. The representational capability of CNNs lies in their effective receptive field, which is proportional to the network depth:

(10) $$R_{l}=R_{l-1}+\left(k_{l}-1\right)\prod_{i=1}^{l-1}s_{i}$$

where $R_{l}$ is the receptive field in layer *l*, $k_{l}$ is the kernel size, and $s_{i}$ is the stride for layer *i* [123]. As the size of the receptive field increases, CNNs can incorporate anatomical and physiological contexts of greater sizes, making it possible to extract hierarchical features at various spatial scales. However, the increase in the receptive field happens in a rather slow way, preventing standard CNNs from capturing the long-range temporal dependencies typical of multi-lead ECG data and cardiac imaging sequences.

In general, the training of a CNN can be represented as the process of minimizing a non-convex empirical risk function using gradient-based optimization algorithms [124]. Therefore, the process of convergence depends greatly on factors such as the network architecture, parameter initialization, optimization algorithm, and statistical properties of the underlying cardiovascular dataset. Problems with class imbalance, inter-patient variability, and differing distributions often lead to decreased performance and calibration on the part of CNNs.

Despite good ability to represent data, CNNs are limited in modeling long-range temporal dependencies because they model only localized spatial relations. Moreover, the representations obtained by CNNs are often not interpretable on their own, requiring additional approaches such as Gradient-weighted Class Activation Mapping (Grad-CAM) or saliency-based methods in order to make them understandable [8]. This has led to the design of recurrent networks, attention models, and graph neural networks that can learn richer temporal and relational representations for reliable cardiovascular artificial intelligence.

### 3.4. Recurrent Neural Networks for Temporal Cardiovascular Modeling

Data related to cardiovascular health have an intrinsic temporal dimension, meaning that the data points carrying diagnostically useful information are distributed over time [125]. This issue is addressed by recurrent neural network variants such as long short-term memory (LSTM) and gated recurrent unit (GRU) networks [126]. These methods have been utilized extensively for analysis of ECG readings, arrhythmia diagnosis, prediction of heart failure, longitudinal risk analysis, and physiological monitoring [6]. To deal with the vanishing gradient problem, LSTM networks employ memory cells regulated by

(11) $$c_{t}=f_{t}⊙c_{t-1}+i_{t}⊙{\tilde{c}}_{t},$$

where $c_{t}$ represents the cell state, $f_{t}$ and $i_{t}$ are the forget and input gates, respectively, and ${\tilde{c}}_{t}$ is the cell state candidate [126]. This gate architecture makes it possible to improve information persistence over long distances.

The training process behind LSTMs involves backpropagation through time (BPTT), in which the gradient recursively flows through the sequence of hidden states:

(12) $$\frac{\partial L}{\partial h_{t}}=\frac{\partial L}{\partial h_{T}}\prod_{k=t+1}^{T}\frac{\partial h_{k}}{\partial h_{k-1}}$$

where L is the training loss and *T* represents the final time step. With longer sequence length, the repeated multiplication of the Jacobian terms can result in vanishing or explosion of gradients, hampering effective learning from long physiological sequences [127]. Gated architectures such as LSTMs significantly help in solving this problem by controlling information flow with the help of memory.

However, there are several issues with contemporary recurrent architectures. First, these networks are sequential in nature, which imposes a limitation on their parallelism and makes them more time-consuming to train. Another problem involves finite memory, which makes them perform poorly on long ECG sequences. These issues have led to the advent of attention-based architectures that can model global dependencies without recursion.

### 3.5. Transformer Architectures for Global Cardiovascular Representation Learning

Transformer architectures have gained popularity in cardiovascular artificial intelligence thanks to their ability to capture long-range dependencies through self-attention as opposed to convolution or recurrent propagation of states [128]. Their global contextual relations have made it possible to achieve improved results in the field of ECG analysis, cardiac imaging, multimodal analysis, generation of clinical reports, and risk prediction for patients [129].

Transformers differ from CNNs and RNNs in not using local receptive fields or sequential processing; instead, they model pairwise interactions between all input units. This makes it possible to learn both local morphological relations and long-range physiological relations at the same time, which is especially convenient when dealing with heterogeneous cardiovascular data.

Scaled dot-product attention is defined as

(13) $$\mathrm{Attention}\left(Q,K,V\right)=\mathrm{Softmax}\left(\frac{QK^{T}}{\sqrt{d_{k}}}\right)V,$$

where *Q*, *K*, and *V* denote the query, key, and value matrices, respectively, and $d_{k}$ is the key dimensionality. The scaling factor $\sqrt{d_{k}}$ stabilizes optimization by preventing excessively large attention scores [130].

Current transformer architectures further improve representation learning through multi-head attention:

(14) $$\mathrm{MultiHead}\left(Q,K,V\right)=\mathrm{Concat}\left({\mathrm{head}}_{1},\ldots ,{\mathrm{head}}_{h}\right)W^{O}$$

where

$${\mathrm{head}}_{i}=\mathrm{Attention}\left(QW_{i}^{Q},KW_{i}^{K},VW_{i}^{V}\right),$$

enabling the network to learn complementary relationships in multiple subspace representations simultaneously and detect different physiological signals. Overall, transformers offer better global representation learning than traditional convolutional and recurrent networks. However, their reliance on big data, computing power, and careful interpretation of attention patterns continues to drive research into effective attention-based learning systems [130].

### 3.6. Graph Neural Networks for Relational Cardiovascular Learning

In many cases, the nature of a cardiovascular disorders is such that it is relational in terms of the anatomical structure, coronary artery system, electrophysiological pathways, and similarity among the patients [131]. This makes graph neural network (GNN) approaches quite appropriate in comparison to Euclidean data-driven approaches such as convolutional, recurrent, or attention-based models [132].

A cardiovascular graph is defined as

G=(V,E),

where $V=\{v_{1},v_{2},\ldots ,v_{n}\}$ represents the nodes corresponding to anatomical or clinical entities and *E* represents the edges corresponding to structural or functional relations between them. The topology of the graph is defined using an adjacency matrix *A*, while the node features are defined using a feature matrix *X* [133].

Graph representation learning is carried out using iterative neighborhood aggregation:

(15) $$H^{\left(l+1\right)}=\sigma ({\hat{D}}^{-\frac{1}{2}}\hat{A}{\hat{D}}^{-\frac{1}{2}}H^{\left(l\right)}W^{\left(l\right)})$$

where $\hat{A}=A+I$ is the adjacency matrix including self-loops, $\hat{D}$ is the degree matrix of $\hat{A}$, $H^{\left(l\right)}$ indicates node embeddings of the current layer, $W^{\left(l\right)}$ is the weight matrix, and σ(·) denotes the activation function [134].

In a more general form, the embedding of node *v* is updated as

(16) $$h_{v}^{\left(l+1\right)}=\phi \left(W\cdot \mathrm{AGG}\left(\{h_{u}^{\left(l\right)}:u\in N\left(v\right)\}\right)\right),$$

where N(v) is the set of neighboring nodes of *v* and AGG(·) stands for an aggregation operator such as mean, sum, or max pooling. Such a message-passing scheme allows each node to incrementally aggregate the structural information in its local neighborhood without disturbing the graph structure of the cardiovascular system [135].

The main strength of GNNs lies in their ability to encode the relational inductive bias into the model and then use it effectively to learn the structures of coronary artery trees, myocardium interactions, cardiac chamber connectivity, and patient similarity graphs. Nevertheless, GNNs still have limitations concerning graph construction, scalability, and oversmoothing, while the scarcity of graph-structured cardiovascular datasets prevents large-scale clinical validation.

In other words, GNNs present a robust mathematical way of encoding the structural and relational information that enhances the representation capabilities of convolutional, recurrent, and transformer networks in trustworthy cardiovascular AI.

### 3.7. Comparative Mathematical Analysis of Deep Learning Architectures

**Figure 8:**
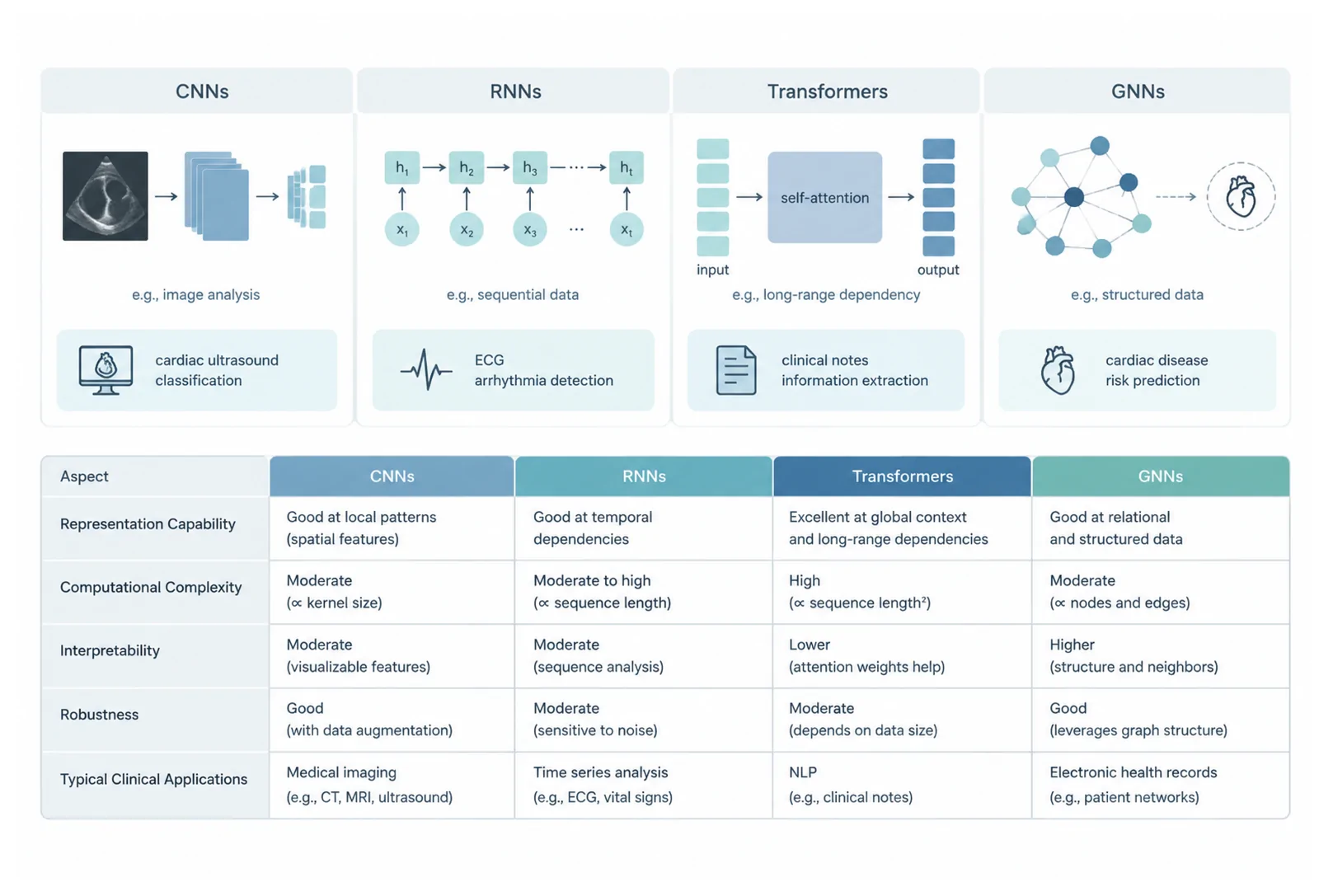
Comparative overview of CNNs, RNN/LSTMs, vision transformers, and graph neural networks, highlighting their mathematical representations, strengths, limitations, computational complexity, interpretability, and clinical applications.

The architectural designs discussed above are distinct in terms of the mathematics involved in their operation and learning of representations, optimization, computational requirements, and clinical feasibility [136]. Figure 8 summarizes these architectural trade-offs across representational capability, computational complexity, interpretability, robustness, and typical clinical applications. Rather than being competing designs, CNNs, RNNs, transformers, and GNNs offer complementary models that are adapted to certain characteristics of cardiovascular data. Therefore, architectural selection involves considering not only prediction ability but also representation capability, computational and optimization efficiency, and other factors.

CNNs achieve localized spatial representation learning using convolutional filters, while RNNs achieve temporal relationship discovery through recursive state updates. On the other hand, transformer models extend sequential modeling by using a self-attention mechanism to perform global context-based learning without the need for recurrence, while GNNs model non-Euclidean structural relationships in cardiovascular data using graph-based message-passing operations. This combination of mathematical approaches is necessary because of the highly varied nature of current cardiovascular data [136].

In this case, architectural design may be thought of as a multi-objective optimization problem:

(17) $$A^{*}=\arg\max_{A\in \Omega }\left[\alpha P+\beta R+\gamma T-\delta C\right]$$

where A is a candidate architecture in the design space Ω, *P* is the predictive performance, *R* is the representational capacity, *T* is the trustworthiness (robustness, interpretability, and fairness), *C* is the computational cost, and α, β, γ, and δ are problem-specific weight factors [8].

There are also important theoretical trade-offs in the architecture selection process. CNNs are computationally efficient, but cannot model long-range dependencies efficiently. RNNs can model temporal relations better than CNNs, but require sequential computation and are subject to gradient instability. Transformers perform much better in global representation learning, but suffer from quadratic increases in computational cost relative to the sequence length, whereas GNNs are efficient in capturing relational patterns but depend heavily on the graph construction process and are subject to scalability and oversmoothing issues. Thus, there is currently no single architecture suitable for all cardiovascular problems.

In general, it is possible to conclude that more trustworthy cardiovascular AI systems will use hybrid and multimodal architectures in the future, combining the advantages of the different architectures mentioned above.

### 3.8. Research Gaps and Future Mathematical Directions

**Figure 9:**
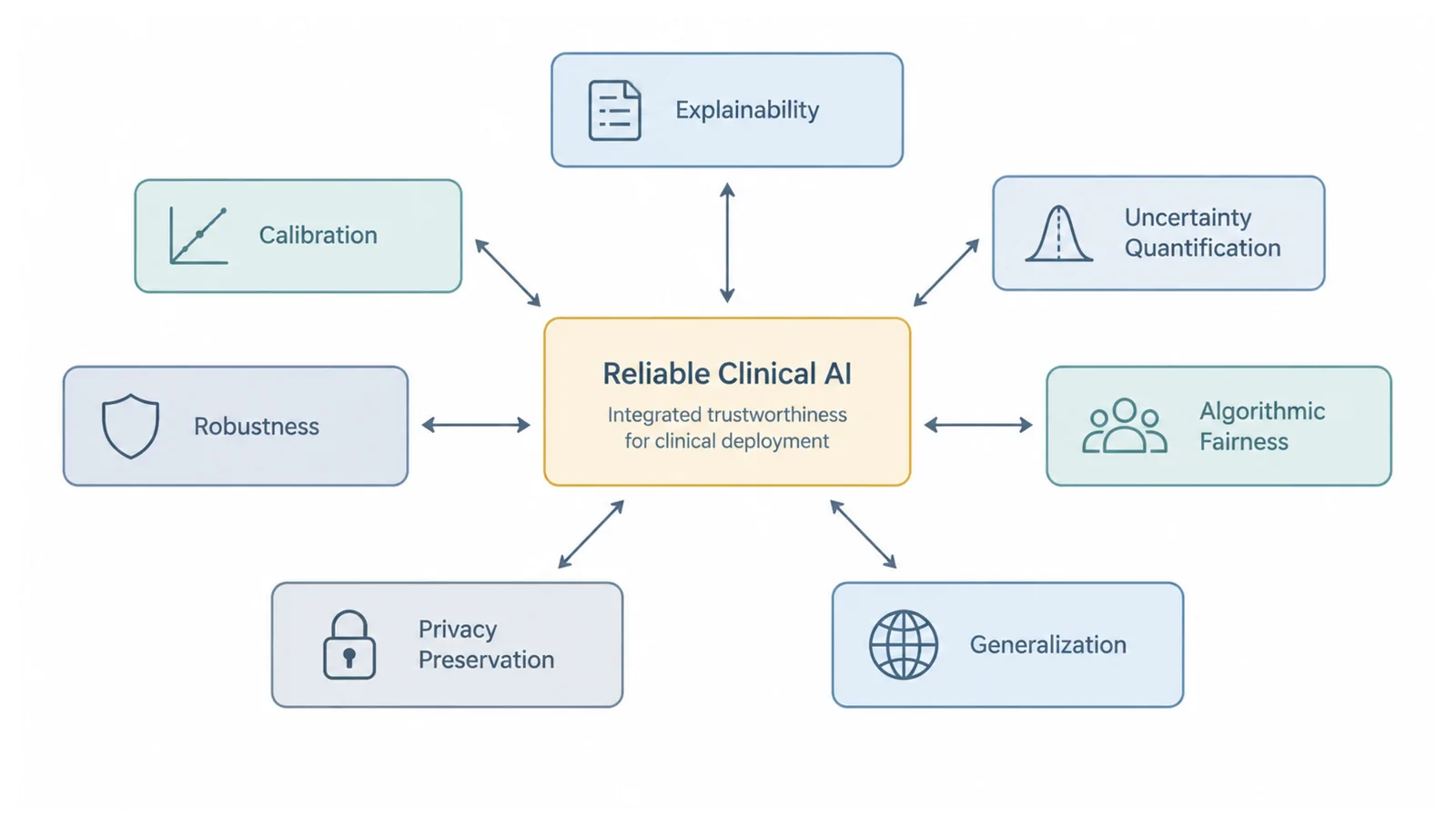
Interactionsamong explainability, calibration, uncertainty, robustness, fairness, privacy, and generalization, showing convergence towards reliable clinical AI systems.

Though many advances have been made in deep learning for cardiovascular problems, there are still many fundamental mathematical problems that prevent further progress in clinical translation. In the reviewed literature, architectural developments have always been ahead of theoretical developments; hence, most of the current models are very effective at making predictions, yet cannot guarantee convergence, robustness, fairness, calibration, and generalization in the case of distribution shift [137]. As illustrated in Figure 9, explainability, calibration, uncertainty, robustness, fairness, and privacy are not independent properties; rather than functioning separately, they jointly interact to determine overall clinical reliability.

It is crucial that both mathematical and predictive performance be seen as equally essential. It is necessary to develop a common theory that would allow for integrating several trustworthiness features into one optimization objective. Currently, most architectures do not have a single optimization objective, meaning that they treat uncertainty estimation, explainability, fairness, robustness, or privacy as separate post hoc features [19].

The next challenge is related to the development of frameworks for multimodal representation learning. Current CV datasets contain combined ECG, echocardiography, cardiac MRI, CT, wearable, EHR, and genomics data; however, only a few models can learn the interactions between different modalities in an efficient way while keeping physiological information in such a representation. Thus, future research should aim at developing mathematically sound frameworks for multimodal representation learning [138].

Scalability should also be considered when thinking about future advances in this domain. Although transformer and GNN architectures significantly increase the capacity to represent complex data, their computational complexity remains high and they require annotated datasets for representation learning. More efficient attention mechanisms, self-supervised learning, foundation models, parameter-efficient adaptation, and federated optimization are among the promising lines in this research direction [17].

A trustworthy model should maintain the same level of performance under various training and test set distributions. This task can be formulated as

(18) $$\min_{\theta }\;E_{\left(x,y\right)∼P_{train}}\;\left[L(f_{\theta }\left(x\right),y)\right]+\lambda D\left(P_{train}^{\theta },P_{test}^{\theta }\right),$$

where $P_{train}^{\theta }$ and $P_{test}^{\theta }$ are the distributions of the learned representations of the training and test data, respectively, while D(·,·) represents a metric for discrepancies among distributions. Minimizing such an objective yields better generalizability of the model to varying patient populations and different scanning protocols [139].

In conclusion, future advances in cardiac imaging AI will require less focus on the development of sophisticated models and more effort put into constructing rigorous learning frameworks based on mathematical theories of optimization, statistical learning, uncertainty estimation, fairness, robustness, and privacy. Such considerations naturally lead to the discussion of trustworthiness principles, which we cover in the next section.

## 4. Trustworthy AI, Fairness, and Privacy in Cardiology

### 4.1. Explainability via Cooperative Game Theory

Explainability is crucial for trustworthy AI in cardiology, as clear and clinically interpretable predictions are needed prior to implementation in clinical settings. Post hoc explanations methods such as SHAP, LIME, and Integrated Gradients offer different mathematical viewpoints on feature attribution for clinical features such as ECG leads, anatomical regions, or physiological biomarkers [140].

SHAP assigns each feature an importance score according to cooperative game theory. The Shapley value of feature *i* is

(19) $$\phi_{i}\left(f,x\right)=\sum_{S\subseteq N\setminus \{i\}}\frac{|S|!(|N|-|S|-1)!}{|N|!}\left[f(S\cup \{i\})-f(S)\right],$$

where *N* denotes the complete feature set and f(S) is the prediction using coalition *S*. This formulation satisfies the axioms of efficiency, symmetry, and additivity, providing theoretically consistent feature attribution [141].

For local interpretability, LIME approximates the original model by a sparse surrogate model through

(20) $$\xi \left(x\right)=\arg\min_{g\in G}\left[L\left(f,g,\pi_{x}\right)+\Omega \left(g\right)\right],$$

where $\pi_{x}$ defines locality around sample *x*, L measures approximation fidelity, and Ω(g) controls model complexity [142].

Gradient-based attribution methods further quantify feature importance through Integrated Gradients:

(21) $$IG_{i}\left(x\right)=\left(x_{i}-x_{i}^{\prime }\right)\int_{0}^{1}\frac{\partial F(x^{\prime }+\alpha \left(x-x^{\prime }\right))}{\partial x_{i}}\;d\alpha$$

where $x^{\prime }$ refers to an initial input vector and F(·) is the predictor model. This definition satisfies the sensitivity and implementation-invariance axioms and mitigates gradient saturation [143].

In sum, all three methods are helpful in explaining cardiovascular predictions. SHAP provides local feature attributions that can be aggregated into global explanations, LIME creates local surrogate models, and Integrated Gradients explain deep neural networks using path-integrated gradients. However, since correlated cardiovascular features such as multi-lead ECG data can result in inconsistent attributions, careful interpretation is required in this case.

### 4.2. Algorithmic Fairness

Fairness in algorithmic systems is critical for the validity of cardiac AI, as any form of bias in the models can result in different prediction performances among various demographic groups [144]. Assessing fairness goes beyond accuracy, as decision-making in AI needs to be consistent among certain protected categories including age, gender, and ethnicity.

One common measure for fairness is the disparate impact (DI) ratio:

(22) $$DI=\frac{P(\hat{Y}=1\;|\;A=\mathrm{unprivileged})}{P(\hat{Y}=1\;|\;A=\mathrm{privileged})}$$

where values close to one indicate statistical parity; the commonly accepted fairness range satisfies 0.8≤DI≤1.25 [145].

To evaluate equality of opportunity, the difference in true positive rates between demographic groups is defined as

(23) $$EOD=TPR_{A=0}-TPR_{A=1},$$

where $TPR=P(\hat{Y}=1|Y=1)$. A value of zero indicates equal opportunity across protected groups [146].

Fairness can be incorporated directly into model optimization through

(24) $$L_{fair}=L_{task}+\lambda L_{bias},$$

where $L_{task}$ is the prediction loss, $L_{bias}$ is a bias term that penalizes demographic discrepancies, and λ balances predictive accuracy and fairness [147].

While there is a growing body of literature focusing on trustworthy AI, it seems that not much work has been done regarding fairness in relation to cardiovascular diseases.

### 4.3. Adversarial Robustness and Stability

Robustness is a core requisite for reliable cardiovascular AI due to the fact that minor perturbations resulting from noisy sensors, variability in data acquisition, and adversarial attacks can cause false clinical predictions. Thus, robust models should remain consistent in their performance under input perturbations.

Robust optimization can be defined as a min–max problem:

(25) $$\min_{\theta }\;E_{\left(x,y\right)}\left[\max_{{∥\delta ∥}_{p}\leq \epsilon }L\left(f_{\theta }\left(x+\delta \right),y\right)\right]$$

where δ represents an adversary perturbation with bound ϵ and L stands for the task loss. In this case, the goal is to obtain parameters that are resilient to worst-case perturbations [148].

The minimum perturbation necessary to change the prediction can be defined as follows:

(26) $$\delta^{*}=\arg\min_{\delta }{∥\delta ∥}_{p}\;s.t.\;f\left(x+\delta \right)\neq f\left(x\right).$$

Equation (26) defines the robustness margin of the classifier. Despite growing interest in adversarial robustness, few cardiovascular studies offer robustness guarantees or test their models in adversarial settings.

### 4.4. Privacy-Preserving Learning

Privacy protection is imperative for reliable cardiac AI due to sensitive data being scattered across various hospitals and healthcare organizations. Privacy-preserving learning techniques protect individual privacy while developing collaborative models.

One of the most popular privacy guarantees is differential privacy (ε-DP), which can be stated as follows:

(27) $$P\;\left[M(D)\in S\right]\leq e^{\varepsilon }P\;\left[M(D^{\prime })\in S\right].$$

When *D* and $D^{\prime }$ differ by one data entry only, M is a randomized algorithm. Here, *S* represents any measurable set of outcomes, while ε represents the privacy parameter. The smaller the value of ε, the more privacy can be ensured [149].

For deep learning problems, differential privacy can be obtained using differentially private stochastic gradient descent (DP-SGD):

(28) $$\theta_{t+1}=\theta_{t}-\frac{\eta }{B}\left(\sum_{i=1}^{B}\mathrm{Clip}\left(g_{i},C\right)+N\left(0,\sigma^{2}C^{2}I\right)\right)$$

where $g_{i}$ is the sample gradient, *C* is the clipping parameter, σ is injected Gaussian noise, and η is the learning rate [150].

Federated learning between multiple organizations usually occurs by federated averaging (FedAvg):

(29) $$\theta^{t+1}=\sum_{k=1}^{K}\frac{n_{k}}{N}\theta_{k}^{t}$$

where $\theta_{k}^{t}$ represents the learned model from client *k*, $n_{k}$ represents its sample size, and $N=\sum_{k=1}^{K}n_{k}$. This approach allows for decentralized learning without sharing any raw patient data [151].

While the number of studies on privacy-preserving learning algorithms has been increasing, deployment in cardiovascular AI systems has not been widespread due to the fact that such techniques come with certain trade-offs.

### 4.5. Unified Trustworthiness Optimization Framework

As can be observed from the earlier subsections, reliable AI for cardiology needs to consider many properties at the same time. However, optimizing each of these objectives separately may lead to conflicting goals; hence, a mathematical framework needs to be established. Section 7 formalizes this joint objective.

## 5. Critical Synthesis and Results

Table 5 contains details on the clinical use cases, data modalities, datasets, validation methods, and trustworthiness dimensions used in the included studies. The numbers and percentages of studies in relation to each of these characteristics were computed based on the extracted information at the study level. Trustworthiness dimensions were categorized based on whether they were clearly assessed and reported, as opposed to being implicitly identified through the model architecture and general discussion by the authors.

The analysis shows that there is an uneven distribution of the dimensions of trustworthiness in the literature on cardiovascular deep learning. Explainability is covered by post hoc methods such as SHAP, LIME, and Integrated Gradients, while calibration, uncertainty estimation, fairness, adversarial robustness, and privacy protection receive less focus. Crucially, the use of an explainability technique does not automatically mean that clinical trustworthiness is demonstrated, and standard performance on test sets does not show robustness or generalization to external populations. This conclusion is drawn from our qualitative analysis of the literature selected for the review; since no full quantitative cross-tabulation of the studies was conducted, the frequency of occurrence of each of the dimensions cannot be considered as a percentage.

### 5.1. RQ1: Quantifying the Representation Deficit

Our results show that while deep learning methods have made tremendous progress in prediction performance in applications to cardiovascular problems, in general there is very limited mathematical characterization of data representation. In the reviewed literature, the large majority of papers use deep learning algorithms on the data in raw or minimally processed form, whereas mathematical characterization of the data representation beyond standard implicit Euclidean feature vectors assumption is scarce.

This issue becomes crucial in the context of cardiovascular artificial intelligence, since physiological data naturally carry geometric characteristics such as time dependency, spatial correlation, anatomical topology, and inter-variable relations. Disregarding these geometric features can cause inconsistency between the actual manifold of the physiological data and the representation assumed by the algorithm.

To quantify this discrepancy in a rigorous fashion, we introduce the representation deficit (RD). We define the RD through the maximum mean discrepancy (MMD) in a reproducing kernel Hilbert space (RKHS). The RD cannot be defined using just the KL-divergence, since this approach requires identical supports. The MMD compares two distributions defined on a common space; the RD is therefore computed in the latent feature space.

Here, X denotes the input physiological data space and Z represents the latent feature space obtained from the model $E_{\theta }:X\to Z$. $Q_{Z}$ denotes the distribution of $E_{\theta }\left(x\right)$ for $x∼P_{X}$, where $P_{X}$ is the physiological data distribution, and $P_{Z}^{*}$ denotes a reference distribution on Z obtained by embedding physiologically annotated reference data. The RD is defined as the MMD between the reference distribution and the learned latent distribution:

(30) $$\mathrm{RD}\left(P_{Z}^{*},Q_{Z}\right)={∥E_{z∼P_{Z}^{*}}\left[\phi \left(z\right)\right]-E_{z∼Q_{Z}}\left[\phi \left(z\right)\right]∥}_{H}$$

where ϕ:Z→H is a feature map to the RKHS H. In practice, when we have finite sample data ${\left\{z_{i}^{*}\right\}}_{i=1}^{n}$ sampled from $P_{Z}^{*}$ and ${\left\{z_{j}\right\}}_{j=1}^{m}$ sampled from $Q_{Z}$, estimation of ${\mathrm{RD}}^{2}$ is performed using a Gaussian kernel $k\left(u,v\right)=\exp\left(-\frac{{∥u-v∥}^{2}}{2\sigma^{2}}\right)$:

(31) $${\hat{\mathrm{RD}}}^{2}=\frac{1}{n(n-1)}\sum_{i\neq j}k\left(z_{i}^{*},z_{j}^{*}\right)+\frac{1}{m(m-1)}\sum_{i\neq j}k\left(z_{i},z_{j}\right)-\frac{2}{nm}\sum_{i,j}k\left(z_{i}^{*},z_{j}\right).$$

A low value for RD means that the model’s internal representation retains the statistical structure of the physiological data, minimizing the chances of finding spurious correlations.

To account for the geometric mismatch, we introduce the manifold alignment error ($\epsilon_{MA}$), which is defined as the Hausdorff distance between the physiological manifold $M_{P}$ and the learned manifold $M_{L}$. Practically, the physiological manifold $M_{P}$ can be obtained using dimensionality reduction methods (such as UMAP and t-SNE) on the data, whereas $M_{L}$ comes from the penultimate layer representations:

(32) $$\epsilon_{MA}\left(M_{P},M_{L}\right)=\max\left(\underset{p\in M_{P}}{\sup}\underset{l\in M_{L}}{\inf}d\left(p,l\right),\underset{l\in M_{L}}{\sup}\underset{p\in M_{P}}{\inf}d\left(l,p\right)\right)$$

where d(·,·) denotes the Euclidean distance in the embedding space. A high value of $\epsilon_{MA}$ may indicate that the classifier uses artifacts for the classification task.

#### Interpretation and Scope of the Representation Deficit

The representation deficit (RD) is suggested as a theoretical metric to quantify the gap between a reference distribution of cardiovascular data and a distribution generated by a representation. The practical calculation of RD requires both distributions to be specified on the same measurable space, as well as a mapping from the observed data to the learned representation. In addition, selection of the metric for measuring the divergence and estimation method should take into account the dimensionality of the representation, the support of the distributions, and the available number of samples. While the Kullback–Leibler divergence offers one possible information-theoretic metric, use of the KL divergence is conditional upon the properties of the distributions, and may not be appropriate if the supports of the two distributions are different.

In the current review, we present the RD as a proposed analytical framework as opposed to a proven empirical metric. In synthesizing the 79 studies for the purpose of identifying methodological trends and limitations in cardiovascular deep learning, RD was not systematically derived from each study. As such, no RD estimates on a corpus level or comparison of RD between individual studies are provided. Numerical interpretation of RD is left for future research, which will require a properly defined representation space and protocol for deriving RD estimates.

To incorporate the discrepancy geometrically, we define the alignment error between the physiological manifold $M_{phys}$ and the manifold learned from the representation $M_{model}$ as follows:

(33) $$\Delta_{M}=d\left(M_{phys},M_{model}\right)$$

where d(·,·) is some appropriate manifold distance metric. Smaller values of $\Delta_{M}$ indicate greater preservation of physiologically relevant structures during the process of representation learning [152].

From our review, it is clear that there is an inherent structural mismatch between the assumed independence of observations in Euclidean space and the structured cardiovascular data in many of the reviewed works. This problem is particularly pronounced in the case of multiple lead ECGs, multimodal cardiac images, and structural modeling using graph theory.

**Figure 10:**
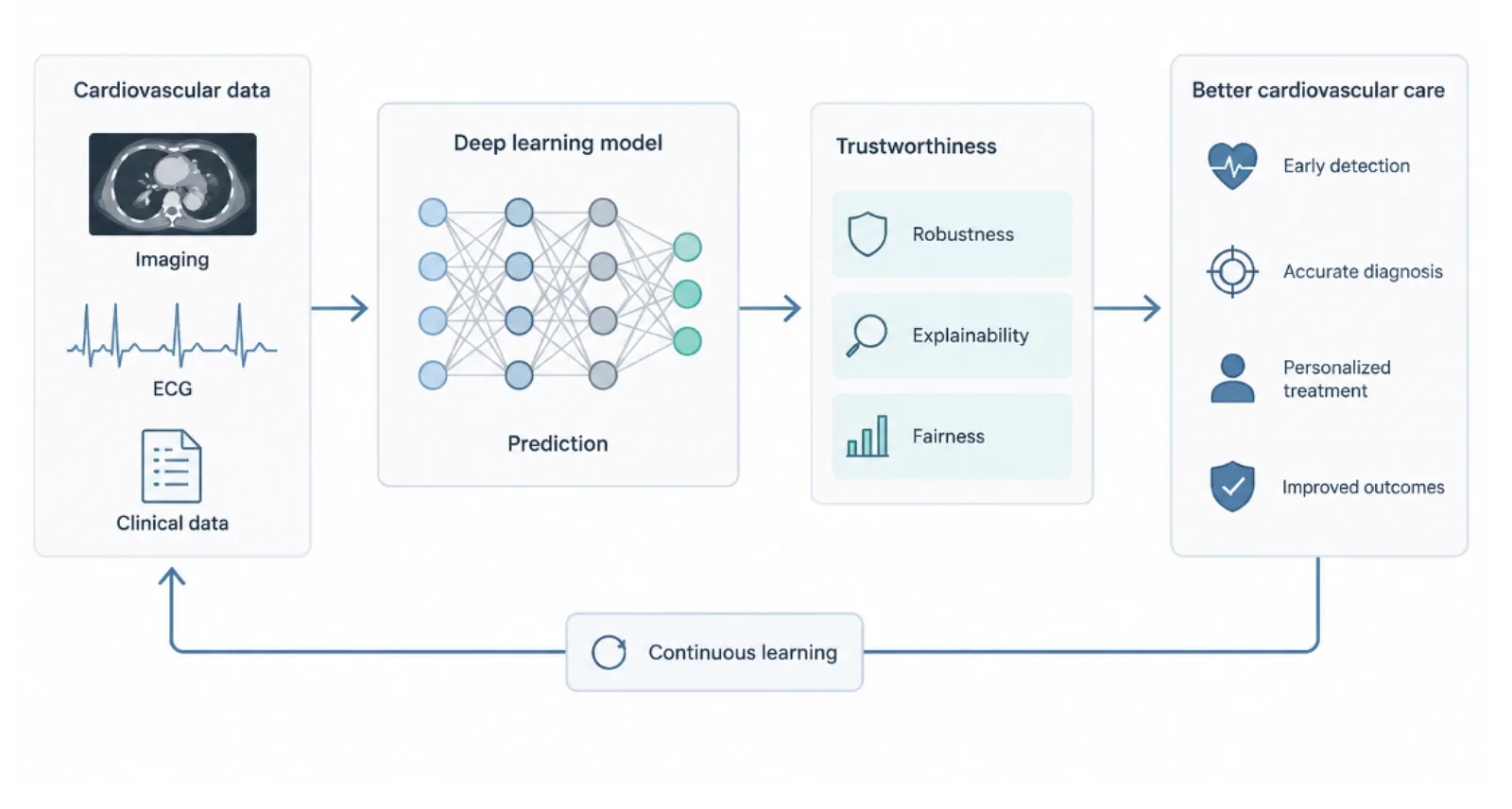
Graphical summary of the proposed mathematical framework integrating evidence synthesis, representation learning, optimization, trustworthiness principles, and clinical deployment.

Generally speaking, the problem of representation mismatch shows that a major limitation of contemporary research on deep learning applications in cardiovascular medicine is that optimization of prediction performance is often performed without taking into account possible inadequacy of the mathematical representation in comparison to actual physiology. Figure 10 summarizes the mathematically-constrained audit pipeline used to synthesize the evidence, spanning representation, optimization, and trustworthiness auditing through to the unified framework.

### 5.2. RQ2: Convergence and Optimization Gaps

The second research question focuses on whether the current body of literature on cardiovascular deep learning captures the dynamics of mathematical optimization and its reproducibility. While most of the papers we reviewed provide extensive details regarding their predictive capabilities, very few provide sufficient detail regarding optimization dynamics, convergence stability, hyperparameter tuning, and training dynamics.

Empirical risk minimization is a common process used in training a deep learning model; the objective is to train the model to minimize the expected difference between predictions and clinical outcomes using parameters θ:

(34) $$\theta^{*}=\arg\min_{\theta }\frac{1}{N}\sum_{i=1}^{N}L\left(f_{\theta }\left(x_{i}\right),y_{i}\right)$$

where L is the task-specific loss function, $f_{\theta }$ is the deep learning algorithm, and $\left(x_{i},y_{i}\right)$ is the input–output pair. However, the highly non-convex nature of the neural optimization problem can lead to many issues, including local minima, saddle points, gradient instability, and initialization sensitivity [153].

To measure the stability of the optimization process in a rigorous way, we suggest a scale-invariant convergence stability metric ($S_{c}$), which is defined by the variance of the norm of the gradient of the loss function in the last epochs of the training process. In contrast to the loss variance, this metric takes into account the size of the gradients.

Let $L_{t}$ be the loss value in epoch *t* and let $\nabla_{\theta }L_{t}$ be the gradient of the loss function with respect to parameters θ. The stability metric is defined as the coefficient of variation of the norms of the gradient over the last *K* epochs:

(35) $$S_{c}=\frac{\sqrt{\frac{1}{K}\sum_{t=T-K+1}^{T}{\left(∥\nabla_{\theta }L_{t}∥-\mu_{G}\right)}^{2}}}{\mu_{G}}$$

where $\mu_{G}=\frac{1}{K}\sum_{t=T-K+1}^{T}∥\nabla_{\theta }L_{t}∥$ is the mean gradient norm and *T* is the total number of training epochs.

**Normalization and Sensitivity:** In order to compare across loss scales (for example, cross-entropy vs. MSE), the stability metric $S_{c}$ employs the coefficient of variation, which is scale-independent. Small values of $S_{c}$ correspond to stable convergence. Values of $S_{c}$ above 0.5 may indicate that the training dynamics are unstable and susceptible to overfitting or saddle point entrapment. The metric helps to visualize the smoothness of the optimization path compared to traditional loss plots, which only provide information about the final loss value.

Finally, reproducibility is another major issue in the field of deep learning in cardiology. There are multiple factors related to the training process that are usually omitted in the reviewed papers, including the learning rate schedule, initialization procedure, optimization method, and techniques for regularization and validation. The reproducibility score (RS) is defined as

(36) $$RS=\frac{1}{\left|\Theta \right|}\sum_{\theta_{i}\in \Theta }I\left(\theta_{i}\in \Theta_{reported}\right),$$

where Θ is the full set of methodological parameters for optimization and $\Theta_{reported}$ is the set of parameters reported by each paper. The indicator function I(·) is equal to one if the parameter is provided and zero otherwise [154].

In conclusion, our research demonstrates that there is inconsistency in the way optimization is reported in scientific literature. Although more complex architectures such as transformers and graph neural networks allow for better representation of information, the optimization process is still strongly dependent on specific optimization settings, datasets, and computing resources. In particular, the absence of reported training processes makes it impossible to compare the results of different studies and independently replicate the results of published papers.

Additionally, specific optimization challenges exist for each type of architecture. Convolutional architectures provide a stable optimization process but encounter problems when the receptive field is small; recurrent architectures are prone to gradient decay; transformers increase computational load due to the attention mechanism; and graph neural networks are sensitive to the topological representation and message-passing depth.

In conclusion, the optimization gap identified in this review calls for future research in cardiovascular AI to not only provide the results of optimization processes but also mathematically describe the convergence behavior of the training process.

**Scope of the illustrative algorithm.** Algorithm 2 gives a simplified operationalization of the proposed TDI through binary indicators of calibration, fairness, robustness, and explainability. It is merely an example, and differs from the continuous formulation in Equation (37). The two are related but distinct specifications. An operational definition that is consistent in terms of components, normalization procedures, and thresholds needs to be defined before either can be used for comparison purposes. The algorithm has not been empirically validated in the current review.

This pipeline is presented as the conceptual procedure underlying the qualitative synthesis in Section 5; its full quantitative execution across the corpus is proposed as future work (see Section 8.2).
**Algorithm 2** Computation of Trustworthiness Deficit Index (TDI)**Input:** Model *f*, calibration error ECE, fairness metric DI, robustness bound $\delta^{*}$, explainability report $E_{rep}$.**Output:** Trustworthiness deficit index TDI.Initialize safety indicators:$$I_{1}=1\left(ECE<\tau_{ece}\right)$$    (Threshold $\tau_{ece}$, e.g., 0.10)$$I_{2}=1\left(0.8\leq DI\leq 1.25\right)$$    (Statistical parity range)$$I_{3}=1\left(\delta^{*}>\epsilon \right)$$    (Robustness margin vs perturbation budget)$$I_{4}=1\left(E_{rep}\;\mathrm{is}\;\mathrm{present}\;\mathrm{and}\;\mathrm{stable}\right)$$    (Explainability presence)Compute compliance score:$$C=\frac{I_{1}+I_{2}+I_{3}+I_{4}}{4}$$Compute deficit:TDI=1−CReturn TDI.

### 5.3. RQ3: The Trustworthiness Deficit Index

**Figure 11:**
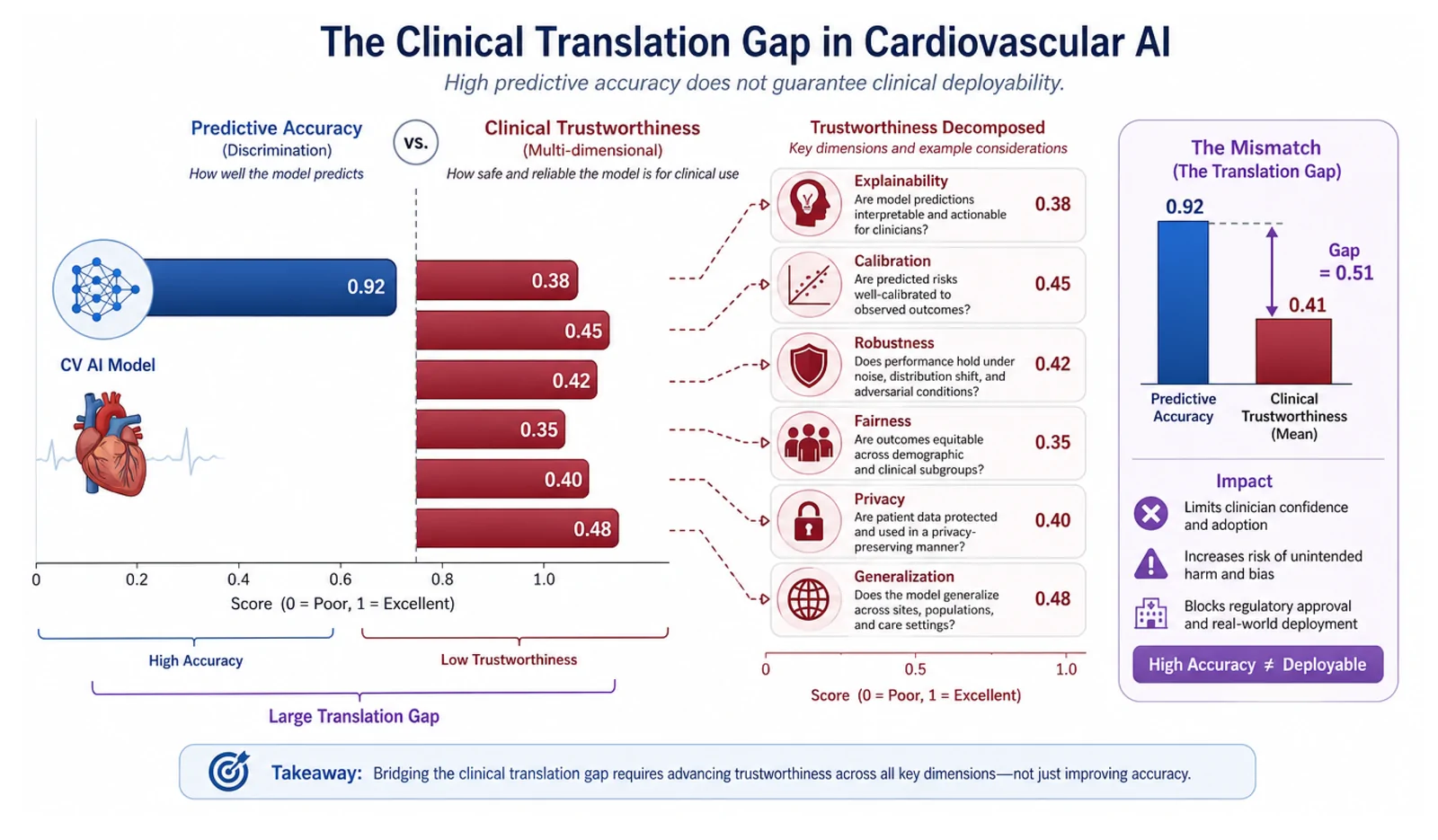
Despite improvements in predictive performance, successful clinical translation also requires explainability, calibration, uncertainty estimation, fairness, robustness, privacy, and external generalization.

The third research question is related to the existence of appropriate mathematical guarantees that can ensure the reliability of existing deep learning systems for clinical use. Despite most of the reviewed papers reporting good prediction results, we found that important features such as calibration, fairness, robustness, and explainability are often evaluated separately or even not considered at all. This discrepancy between high predictive power and relatively low trustworthiness scores is demonstrated in Figure 11.

To evaluate the extent of this problem, we propose a new metric, the trustworthiness deficit index (TDI), which measures the gap between the required trustworthiness features of the system and the real model characteristics:

(37) $$TDI=1-\frac{\sum_{j=1}^{4}w_{j}C_{j}}{\sum_{j=1}^{4}w_{j}},$$

where $C_{j}$ is the normalized compliance value of the *j*-th criterion: calibration ($C_{cal}$), fairness ($C_{fair}$), robustness ($C_{rob}$), and explainability ($C_{exp}$). Without domain-specific preference weights, we adopt equal weights ($w_{j}=1$) in order to ensure neutrality under different clinical settings. Each $C_{j}$ can be binary or continuous within [0,1] depending on whether the paper satisfies certain safety conditions (for example, ECE<0.10 for calibration). A value of TDI=0 corresponds to a perfectly compliant paper, while higher values indicate more deficiencies in clinical trustworthiness.

#### Operational Specifications and Limitations

To make sure that the TDI framework is an effective auditing framework, the following operational specifications are made:

- **Normalization and Thresholds:** Compliance scores are normalized according to conventional thresholds from the literature; for example, fairness is considered compliant when the disparate impact ratio DI∈[0.8,1.25], while calibration is considered compliant when ECE<0.10.
- **Missing Assessment Handling:** In the case of some dimension (e.g., robustness) not being assessed in a particular study, it is considered non-compliant ($C_{j}=0$); if a partial score is preferred, it can instead be excluded from the denominator of the compliance score.
- **Comparability:** Although the aggregated TDI score provides a single measure, it can miss certain weaknesses. Thus, it is recommended to report individual scores along with the overall score. The TDI index is constructed in order to be comparable across different diseases and modalities, as it uses standardized dimensionless compliance ratios instead of performance metrics.
- **Exploratory Nature and Applicability:** It must be clearly stated that our introduction of the TDI index in this review is intended merely to serve as a conceptual and exploratory audit tool; the use and empirical testing of the index on diverse cardiovascular datasets is not within the purview of this review, and represents a research path that needs to be explored in future work. This review provides the theoretical basis for the index, which should be viewed only as an exploratory vocabulary.

The measure of calibration consistency is the expected calibration error (ECE):

(38) $$ECE=\sum_{m=1}^{M}\frac{|B_{m}|}{N}\left|acc\left(B_{m}\right)-conf\left(B_{m}\right)\right|$$

where $B_{m}$ is the *m*-th confidence interval bin, $acc(B_{m})$ is the empirical accuracy, and $conf(B_{m})$ is the average predicted confidence. Lower ECE values indicate better agreement between the predicted probabilities and reality. Based on our qualitative assessment, we argue that many deep learning papers with applications to cardiovascular studies tend to emphasize prediction accuracy at the expense of trustworthiness-related aspects. More specifically, analyses of calibration and fairness do not receive enough attention, whereas evaluations of robustness are limited to performance on test sets [155].

However, it should be emphasized that the measures such as RD, $\Delta_{M}$, RS, and TDI introduced above are supposed to work as auditing tools. We did not exhaustively compute scores for the 79 analyzed studies. Computation of the above measures would include the creation of calibration curves, analyzing fairness breakdowns, and adversarial testing, which is not provided in the majority of the existing literature related to deep learning models in cardiology. Therefore, our qualitative synthesis shows that such measures should be considered as an auditing vocabulary for the future, when reporting standards become more uniform. Table 6 provides a qualitative picture of the attention paid to the mentioned trustworthiness dimensions in the reviewed literature.

This evidence suggests that predictive accuracy alone is insufficient for practical uses. In order to make AI for cardiovascular disease clinically useful, it needs to be capable of highly accurate probabilistic predictions while simultaneously providing fairness, stability, and explainability. The proposed TDI approach can act as an umbrella theory for auditing and identifying crucial gaps in theis domain.

### 5.4. RQ4: Open Mathematical Challenges

While deep learning approaches to cardiovascular science have shown high predictive accuracy, there are still mathematical issues that need to be resolved before they can to reach the level of reliable clinical application. From our analysis, four significant areas of study need more research: uncertainty quantification, privacy-preserving optimization, physics-informed modeling, and unified trustworthy objectives.

**Uncertainty Quantification:** Existing methods mostly produce deterministic predictions without assessing their confidence reliability. Bayesian deep learning frameworks provide an elegant solution in which the model parameters are treated probabilistically instead of deterministically:

(39) $$p\left(y|x,D\right)=\int p\left(y|x,w\right)p\left(w|D\right)dw\approx \frac{1}{T}\sum_{t=1}^{T}p\left(y|x,w_{t}\right)$$

where p(w|D) denotes the posterior probability of the model’s parameters, with the Monte Carlo approximation quantifying predictive uncertainty using several stochastic forward passes. Uncertainty-aware models are necessary to detect unreliable predictions in risky decision-making situations involving cardiovascular disease [156].

**Federated Learning and Non-IID Optimization:** The multi-center setting of CVD datasets poses challenges around privacy restrictions and non-IID samples from different centers. Federated learning allows for joint model training without aggregating data in one place. The global objective function may be written as follows:

(40) $$\min_{\theta }\sum_{k=1}^{K}\frac{n_{k}}{N}L_{k}\left(\theta \right)$$

where *K* is the number of participating clinical sites, $n_{k}$ is the local sample size, and $L_{k}$ is the local objective function. However, convergence can be a problem under non-independent and identically distributed (non-IID) cardiovascular data, necessitating advanced techniques for aggregating such data which exceed the capabilities of FedAvg [151].

**Physically Constrained Cardiovascular Learning:** In the future, any trustworthy system should take physiological constraints into account during training.

**Unified Trustworthy Optimization:** Finally, any future cardiovascular AI model should include performance optimization while concurrently meeting the relevant trustworthiness criteria. Therefore, future cardiovascular artificial intelligence research should focus not just on creating more sophisticated architectures but on developing learning algorithms with a mathematical basis together with reliability guarantees under the conditions of uncertainty and distribution shifts.

## 6. Discussion

### 6.1. Architectural Trade-Offs and Clinical Reliability

Our review highlights that the selection of a deep learning architecture greatly affects not only the predictive power of the model but also the inherent characteristics of the model’s trustworthiness, such as robustness, interpretability, and generalization to shifts in distributions.

**Convolutional Neural Networks (CNNs):** From the evidence gathered in the literature review, it is clear that CNNs continue to be the primary architecture for ECGs and cardiac images because of their translation-invariance and local feature extraction properties [100]. From a mathematical perspective, the properties of the CNNs, namely, their sparsity and sharing of parameters, act as an implicit regularizer, allowing the network to remain robust against the small spatial deformations and noise present in physiological signals [8]. Nevertheless, our review shows that CNNs have difficulties in handling long-term dependencies present in multi-lead ECGs, and can also misclassify arrhythmias requiring a more global view [100]. Moreover, although CNNs are computationally efficient, they are black boxes, and as such need to be explained using techniques such as Grad-CAM, which can be unstable when used on correlated cardiovascular features [21].

**Recurrent Neural Networks (RNNs/LSTMs):** Research on using LSTMs for predicting risks and heart failure prognostics has shown that LSTMs have better capacity to capture temporal dynamics [125]. Gating functions in LSTMs address the vanishing gradient problem and allow for the storage of important information about patient history. On the other hand, the sequential design of RNNs makes them sensitive to initialization and prone to gradient instabilities during training, as stated in several of the reviewed articles [157]. More importantly, RNNs cannot generalize with respect to sampling rate or time between clinical events, which is a common problem in EHRs [107].

**Transformers:** Recent research involving vision transformers (ViTs) and ECG transformers has shown state-of-the-art results in terms of capturing global dependencies and multimodal interaction [129]. Dynamic weighting of relevant physiological fragments is made possible through the self-attention mechanism, which may prove to be a more accurate foundation for explainability than static convolutional filters. However, in our analysis we find that there is a substantial “data hunger” issue with transformers; they tend to overfit on smaller and single center cardiovascular datasets, resulting in poor calibration and lack of robustness to distributional shifts [130].

**Graph Neural Networks (GNNs):** The use of GNNs in the representation of anatomical structures (coronary artery trees) and patient similarity graphs is gaining momentum [111]. Thanks to their capacity to incorporate relational inductive biases, GNNs can provide a mathematically sound framework for non-Euclidean cardiac data representation. It has been shown that GNNs have the potential to promote fairness through their incorporation of demographic relations in patient graphs [121]. However, the performance of GNNs is greatly dependent on the quality of the graph creation process.

**Generalization and Distributional Shifts:** Another important observation related to all architectures is the absence of thorough external validation. Most of the reviewed studies relied on internal validation, which does not take into account the variance of patients or possible distribution shifts on-site, such as different ECG machines or MRI scanners [12]. This issue dramatically reduces the clinical value of such research, as models that work well on test datasets may not perform well in different clinical settings. Our analysis indicates that generalization cannot be achieved through architectural approaches alone; optimization for invariance to domains is needed as well.

In conclusion, while each architecture has its mathematical benefits, there is currently no single architecture that would allow for the development of reliable cardiac AI tools. The tension between prediction accuracy and clinical reliability is an essential question that requires moving from simple model selection based on accuracy to a more complex evaluation framework.

### 6.2. Critical Limitations of Trustworthiness Metrics

Although the reviewed literature increasingly recognizes the significance of trustworthiness, our analysis shows that many of the suggested metrics and approaches are riddled with contextual dependency and theoretical weaknesses that are commonly ignored by clinical AI researchers.

**Calibration and Dependence on the Evaluation Dataset:** Calibration measures such as the expected calibration error (ECE) are very dependent on the evaluation process. Prior work shows that the ECE scores of a model can differ greatly depending on the number of bins used in histogram binning and the distribution of the dataset used for evaluation [155]. For instance, the same model may be successfully calibrated on an internally balanced test dataset, then show poor calibration performance when evaluated on an externally diverse dataset with varying prevalence rates.

**Context-Based Fairness:** Fairness measures are highly dependent on the protected attributes (such as age, gender, race) as well as on the particular clinical setting. For example, a certain model may be statistically fair according to the gender attribute for arrhythmia identification while not being statistically fair for heart failure prediction due to the natural biological differences in disease manifestation [144]. We found that the majority of papers in our review rely on a single fairness measure such as disparate impact, without taking into account possible trade-offs between different fairness definitions.

**Robustness and Distributional Shifts:** Robustness evaluations in the surveyed literature are restricted to additive perturbations or small geometric transformations. In contrast, clinical use cases require robustness against distributional shifts involving different imaging protocols, different manufacturers of sensors, and varying patient demographics. We observed that robustness to a certain kind of perturbation (for example, Gaussian noise) does not imply robustness to other types (such as adversarial perturbations or domain shifts) [148]. The magnitude of the shift is also relevant; some models might be robust to small perturbations but fail under large distributional shifts.

**Stability and Faithfulness of Explainability:** Whereas numerous papers use post hoc explainability approaches such as Grad-CAM or SHAP, our critical synthesis reveals that visual explanations are often considered clinically relevant without any validation. According to recent research, such techniques can be unstable and provide very different attributions for similar input images; in addition, they can be unfaithful and may not represent the real decision-making process of the model [21]. Not estimating the stability and faithfulness of explanations can lead clinicians to believe in artifacts instead of real physiological characteristics.

**Privacy Guarantees vs. System Security:** Finally, although techniques such as differential privacy (DP) and federated learning (FL) offer robust mathematical guarantees on the privacy of the training phase, they do not necessarily guarantee the security of the resulting system. A model trained using DP is still susceptible to model inversion attacks and membership inference attacks if not secured properly at the inference phase [63]. Additionally, although FL solves data sharing problems, it exposes the system to new risks around model poisoning and communication security. Our literature review highlights that privacy-preserving training should be coupled with secure deployment in order to ensure overall clinical safety.

To summarize, there is a need for a more nuanced approach to the existing metrics in the current state of trustworthy cardiac AI. Future studies should go beyond presenting metrics as simple single value numbers and perform sensitivity analyses to consider factors such as dataset properties, clinical context, and the nature of possible threats.

**Figure 12:**
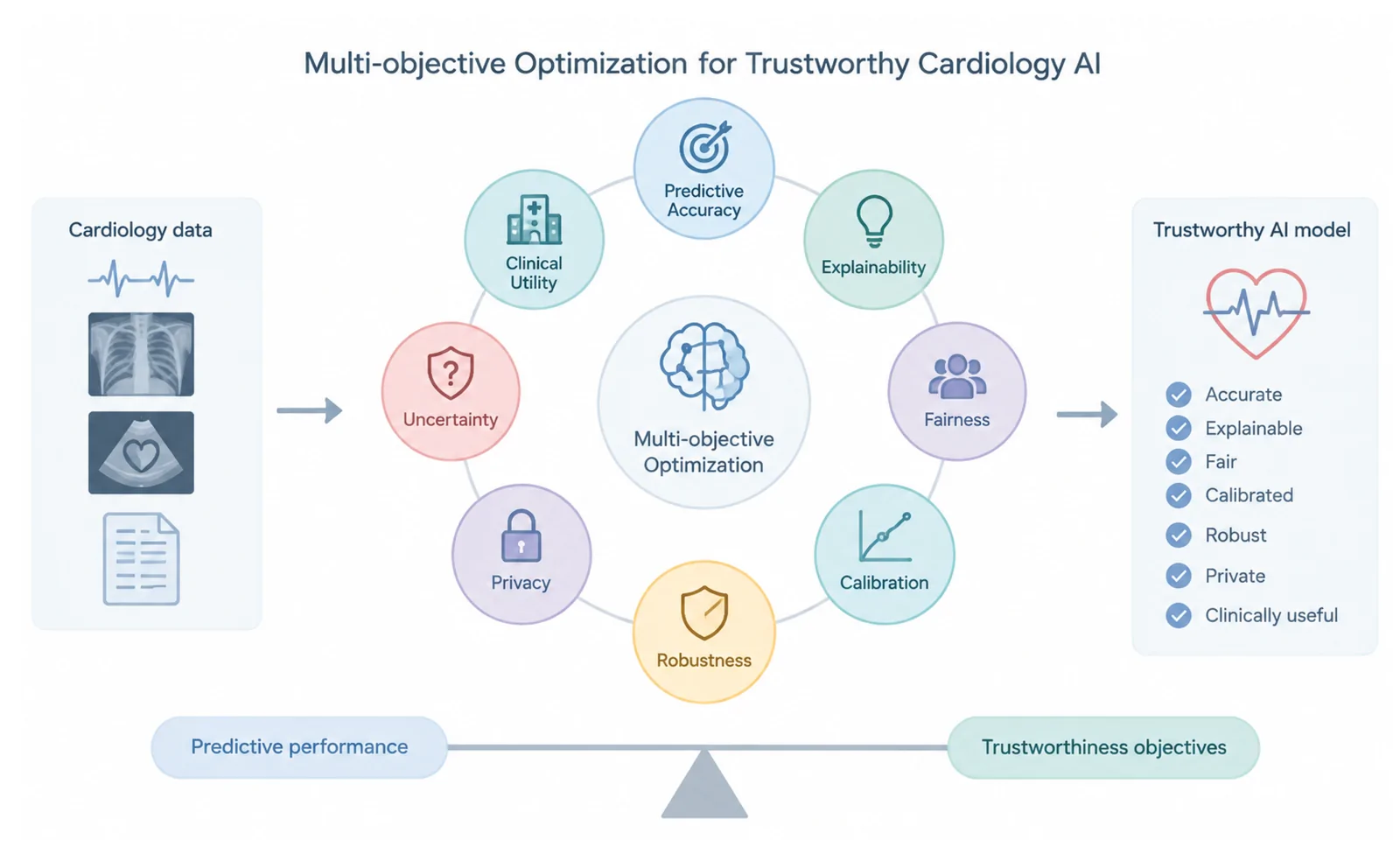
Conceptualillustration of the balance between predictive performance and explainability, robustness, calibration, uncertainty, fairness, privacy, and clinical utility.

It is clear from the current review that the fast evolution of deep learning in cardiovascular medicine has led to significant improvements in terms of prediction accuracy on various applications in cardiology, such as electrocardiography, cardiac imaging, signal processing, and multimodal decision-making [158]. Figure 12 conceptually illustrates this multi-objective trade-off space, where predictive accuracy must be balanced against explainability, calibration, robustness, fairness, and privacy along a Pareto front of clinically viable solutions. Nevertheless, the systematization performed within this paper indicates that while the methodology has undergone substantial development, the mathematical foundation of trustworthiness has not followed. While numerous modern papers boast of remarkable prediction accuracy, far fewer can justify their claims using concepts such as model calibration, uncertainty estimation, robustness evaluation, fairness considerations, and privacy-preserving learning. The key contributions of the present review include unifying all of these seemingly independent research tracks under one mathematical approach. Instead of regarding the representation learning, optimization, explainability, uncertainty, robustness, fairness, and privacy as separate parts, the framework presented above highlights the mathematical connections between them across the whole pipeline of deep learning [19]. The key point here is that trustworthy cardiovascular artificial intelligence should be seen not as a set of post hoc evaluation methods but as an optimization problem in which prediction accuracy and clinical trustworthiness are maximized together.

The results of the present review emphasize that the evaluation of cardiovascular deep learning models should not be limited to their ability to make predictions. Given the connection of representation learning and optimization with trustworthiness criteria, the presented perspective can serve as a starting point for uncovering research gaps and developing further studies.

#### 6.2.1. Conceptual Contribution of the Proposed Auditing Constructs

We introduce the RD and TDI as potential constructs that can be used to organize future audits of representation adequacy and multidimensional trustworthiness in cardiovascular deep learning. The RD represents a kernel-based construct, and is suggested as a perspective on representation discrepancy, while TDI represents a potential way to summarize several different trustworthiness dimensions. The role of these constructs in the present research is purely theoretical, as none of them was systematically estimated or empirically validated on the 79-study corpus; thus, their operational definitions, estimations, thresholding, and validation remain open questions. These constructs should be considered as a starting point for future methodological research rather than as already-established measures for ranking clinical AI systems.

**Overcoming Heterogeneity Using Narrative Synthesis:** We recognize the high levels of heterogeneity in both methodology and data across the 79 studies we analyzed, and how this prevents the use of quantitative meta-analysis. Instead of seeing this as an obstacle, however, our mathematical model provides a way to use such heterogeneity as an opportunity to conduct narrative synthesis. Specifically, by using the proposed RD and TDI metrics to examine how different architecture types (CNNs, RNNs, transformers, and GNNs) tackle particular aspects of trustworthiness, we outline a structure for a future cross-tabulation that would be impossible with statistics alone.

Based on the evidence derived from the literature review, existing research is still heavily architecture-oriented [159]. A great deal of work has gone into creating ever more advanced models using convolutional, recurrent, transformer, and graph neural networks, while comparatively little has been done on understanding how these models behave under changes in distribution and diverse populations. With cardiovascular datasets becoming larger and more diverse owing to imaging, physiologic measures, EHRs, wearables, and genomics, future advances will be dependent on mathematically sound multimodal learning approaches that can ensure both predictive and model accuracy. The next critical finding based on the analysis of available literature is the fact that many dimensions of trustworthiness are often assessed separately. Explainability, calibration, uncertainty quantification, fairness, robustness, and privacy are often examined as independent goals through separate evaluation protocols. The mathematical approach we introduce here overcomes these disadvantage by offering a shared theoretical foundation that unifies these complementary characteristics. This kind of integration can be instrumental in developing standard evaluation protocols for future cardiovascular deep learning research.

However, the aforementioned advantages also come with some remaining issues. Public cardiovascular disease datasets are usually characterized by demographic imbalance, lack of external validation, and significant variance in acquisition protocols, which makes the generalizability of models questionable [6]. In addition, the literature lacks rigorous mathematical proofs related to robustness, calibration, and uncertainty of models. Solving these issues will require the joint efforts of machine learning specialists, mathematicians, and clinicians [160]. In conclusion, this review goes further than an ordinary survey of the current algorithmic methods by presenting a coherent mathematical synthesis to promote the construction of trustworthy deep learning for cardiological practice. We expect that our framework will be valuable for the future development, evaluation, and safe implementation of artificial intelligence systems able to facilitate decision-making in the context of cardiology.

#### 6.2.2. Limitations of the Proposed Auditing Constructs

Even though RD and TDI can serve as a theoretical basis for discussions regarding representation adequacy and multidimensional trustworthiness, it is important to note that they cannot be considered empirically proven measures based on the findings of this review. First, the differences between the 79 reviewed studies, together with differences in their evaluation measures and the availability of information, did not allow for a quantitative numerical evaluation of our proposed metrics. Further research is needed in order to operationalize RD by providing representation mappings and reproducible divergence estimation, as well as to clearly define TDI components, normalization, and clinically meaningful thresholds. Both measures still need empirical validation through appropriate sensitivity analysis and independent validation on datasets.

## 7. The Unified Mathematical Framework for Trustworthy Cardiac AI

**Figure 13:**
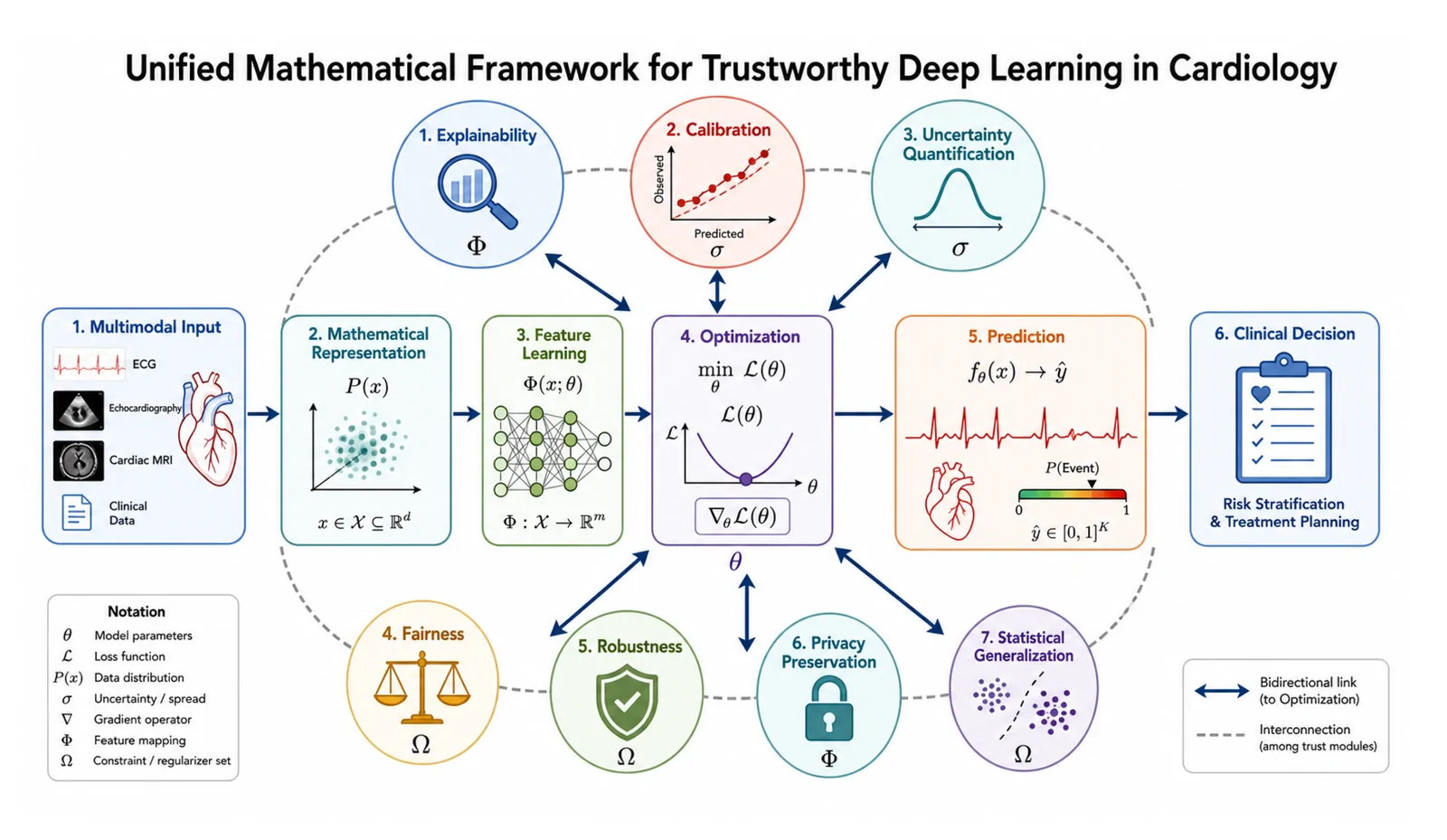
The proposed framework integrates representation learning, optimization, prediction, and clinical decision-making while jointly enforcing explainability, calibration, uncertainty quantification, fairness, robustness, privacy, and statistical generalization.

It is clear from the foregoing discussion that credible cardiovascular artificial intelligence calls for the consideration of data representation, optimization dynamics, and reliability requirements in tandem. In light of the deficiencies in current research, we have developed a mathematical framework for synthesis which will help to integrate these aspects together in an auditable learning pipeline [161]. This framework does not offer a new architecture, only a conceptual framework. Figure 13 depicts the proposed framework, which integrates representation learning, optimization, and prediction with joint enforcement of calibration, uncertainty, fairness, robustness, and privacy constraints.

### 7.1. Multimodal Data Space

Cardiovascular information is highly varied, consisting of time-variant physiologic signals, medical imaging data, structured clinical information, and relational anatomy models. Thus, it follows that the domain of the input data can be characterized by a collection of mathematical spaces:

(41) $$X=X_{ECG}\cup X_{IMG}\cup X_{EHR}\cup X_{GRAPH}$$

where each component represents a distinct physiological information space requiring appropriate representation learning strategies [162]. Measurement variability is incorporated as

(42) x(t)=s(t)+ϵ,ϵ∼N(0,Σ),

where Σ represents the noise covariance matrix [163].

### 7.2. Optimization Dynamics and Learning Objectives

Training cardiovascular deep learning models involves optimization over highly non-convex parameter spaces. Adaptive optimization methods such as Adam can be used to update model parameters according to

(43) $$\theta_{t+1}=\theta_{t}-\frac{\eta }{\sqrt{{\hat{v}}_{t}}+\epsilon }{\hat{m}}_{t},$$

where ${\hat{m}}_{t}$ and ${\hat{v}}_{t}$ denote bias-corrected first- and second-order moment estimates [164].

To incorporate clinical reliability objectives, the optimization process can be extended beyond predictive accuracy:

(44) $$\begin{array}{cc} \theta^{*}=\arg\min_{\theta } & (L_{task}+\lambda_{u}L_{uncertainty}+\lambda_{f}L_{fairness}\\ & \;\;+\lambda_{r}L_{robustness}+\lambda_{p}L_{privacy}), \end{array}$$

where the additional regularization terms enforce desirable properties for clinical deployment [155].

### 7.3. Unified Trustworthiness Constraints

The suggested model takes into account three key components of trustworthiness: explainability, fairness, and robustness. The component of explainability is based on attributions through algorithms such as SHAP, which make it possible to recognize clinically meaningful features behind the predictions. Fairness constraints provide similar performance for various demographic groups by using statistical parity.

The important relationship between a model’s robustness and smoothness can be expressed through Lipschitz continuity. The Lipschitz continuity of the classifier f(x) with Lipschitz constant *K* is

(45) $$∥f\left(x_{1}\right)-f\left(x_{2}\right)∥\leq K∥x_{1}-x_{2}∥.$$

A lower Lipschitz constant implies smoother decision boundaries and improved resistance to perturbations [165].

#### (1) Proposition: Robustness–Lipschitz Relationship

For a binary classifier with prediction margin

(46) m(x)=|f(x)−0.5|,

the minimum perturbation required to alter the prediction satisfies

(47) $$\delta^{*}\left(x\right)\geq \frac{m(x)}{K}.$$

This dependence highlights the importance of enlarging decision margins and reducing sensitivity as key strategies to increase robustness of clinical AI [166].

### 7.4. Illustrative TDI Example

In order to show how this framework can be applied, we will look at a fictional deep learning study that suggests the use of a CNN for detecting atrial fibrillation (AF) using 12-lead ECGs. This will help us go through the process of extracting evidence and scoring components.

**Point of Clarification.** The following section presents a purely hypothetical exercise involving fictional data, and is intended only to illustrate the process involved in conducting an exploratory TDI analysis. No study examined in the reviewed literature has been evaluated using this specific methodology. The proposed TDI and RD metrics have not yet been empirically validated.

**Step 1: Evidence Extraction.** From the hypothetical study, we extract the following reported metrics:

- **Performance:** Accuracy = 98%, Sensitivity = 97%.
- **Calibration:** Expected calibration error (ECE) = 0.12.
- **Fairness:** No subgroup analyses for age or gender.
- **Robustness:** Only tested on internal hold-out dataset; no adversarial or noise perturbation tests conducted.
- **Explainability:** Grad-CAM visualization provided for ten random samples.
- **Representation:** Raw ECG signals; no manifold alignment analysis provided.

**Step 2: Component Scoring and Missing Information Treatment.** Using the thresholds defined in Section 5.3 and Algorithm 2:

- **Calibration ($C_{cal}$):** $ECE=0.12>\tau_{ece}$ (0.10); therefore, $C_{cal}=0$.
- **Fairness ($C_{fair}$):** Subgroup analysis is not provided; based on our conservative assumption used to ensure transparency, this lack of evidence means non-compliance, so $C_{fair}=0$.
- **Robustness ($C_{rob}$):** No external or adversarial testing is conducted; therefore, $C_{rob}=0$.
- **Explainability ($C_{exp}$):** Grad-CAM is mentioned; however, since no stability scores are provided, we give a half-point score of $C_{exp}=0.5$.

**Step 3: TDI Calculation and Interpretation.** Substituting these values into the four-component TDI formulation (Equation (37)) gives

(48) $$\begin{array}{cc} TDI & =1-\frac{1}{4}\left(C_{cal}+C_{fair}+C_{rob}+C_{exp}\right)\\ \; & =1-\frac{1}{4}\left(0+0+0+0.5\right)\\ \; & =1-0.125\\ \; & =0.875. \end{array}$$

A TDI value of 0.875 represents a severe trustworthiness deficit. Although the model is highly accurate (98%), it does not succeed at calibration, fairness, and robustness. In terms of clinical auditing, such a result would mean that the model is not ready for use and needs further improvement in terms of uncertainty and bias.

**Step 4: Representation Deficit (RD) Assessment** Since the illustration study does not provide latent space distributions or manifold alignments, we cannot calculate the RD numerically; however, qualitatively, the use of raw ECGs without accounting for lead inter-dependencies suggests a potentially high RD. This highlights the need for future studies to report representation metrics alongside performance.

This illustrative example clarifies that the TDI is not merely a performance metric, but an audit score that penalizes missing evidence and poor calibration, thereby distinguishing it from traditional accuracy-based evaluations.

### 7.5. Framework Summary

The unified framework lays out a mathematical approach by which reliable cardiac AI can be developed through an optimization problem subject to the constraints of physiological modeling, learning stability, and clinical validity requirements. Through the unification of multiple concepts such as representation learning, optimization, uncertainty, fairness, and robustness, our unified framework allows for the development of deep learning methods that are not only accurate but also interpretable and clinically applicable.

### 7.6. Integration of Research Questions, Evidence, and Framework

The unified framework proposed in this paper is a conceptual framework derived from the findings collected in the course of answering our four research questions (RQs).

**From RQs to Framework Components:**

- **RQ1 (Representation):** We discovered a major representation deficit problem in the reviewed literature. This is due to models lacking the ability to capture the geometry of cardiovascular datasets. This observation prompted us to design the proposed RD metric (Section 5.1), which can measure the difference between physiological manifolds and latent spaces.
- **RQ2 (Optimization):** Given the research gaps related to convergence evaluation and reproducibility, we developed two metrics to ensure that optimization processes are auditable: the convergence stability metric ($S_{c}$) and the reproducibility score (RS) (Section 5.2).
- **RQ3 (Trustworthiness):** Fragmented evaluation of calibration, fairness, robustness, and explainability of models across the 79 reviewed studies required an auditing framework that could address these challenges. Thus, we developed the trustworthiness deficit index (TDI) (Section 5.3).
- **RQ4 (Challenges):** Open problems identified in this paper, including distributional shift and privacy, influenced the design of our proposed unified optimization framework (Algorithm 3).

**Assumptions and Interactions:** The proposed framework rests on three main assumptions: first, trustworthiness is multidimensional, and cannot be measured only in terms of accuracy; second, mathematical precision is needed in order to make trustworthiness auditable; and third, absence of evidence of trustworthiness indicates deficiency. The above components work together synergistically: high RD indicates bad representativeness, which may coincide with unstable optimization (high $S_{c}$) and low trustworthiness (high TDI); in other words, the framework provides a holistic way to examine a model’s reliability.

**Difference Between Literature Findings and the Constructed Framework:** It is important to differentiate between findings that we obtained from the literature and constructs that we have introduced in this paper. The literature findings (e.g., the limited use of external validation) are empirical findings from the systematic review, the proposed constructs (RD, TDI, and $S_{c}$) are new mathematical frameworks proposed in this paper to solve the identified problems. Although the constructs are based on the problems identified during our literature review, they have not yet been empirically proven due to the many differences between the 79 reviewed studies (see Section 8.2).
**Algorithm 3** UnifiedOptimization Framework for Trustworthy Cardiac AI**Require:** 
Cardiac dataset D, model parameters θ**Ensure:** 
Optimized trustworthy model $\theta^{*}$  1:Represent cardiovascular data:X→{tensor,temporal,graph}  2:Initialize model parameters θ  3:**while** not converged **do**  4:      Compute prediction loss:$$L_{task}$$  5:      Calculate trust constraints:(49)$$\begin{array}{cc} L_{\mathrm{total}}= & L_{\mathrm{task}}+\lambda_{u}L_{\mathrm{uncertainty}}\;\\ & +\lambda_{f}L_{\mathrm{fairness}}+\lambda_{r}L_{\mathrm{robustness}}. \end{array}$$  6:      Update parameters:$$\theta_{t+1}=\theta_{t}-\eta \nabla_{\theta }L_{total}$$  7:**end while**   **return** $\theta^{*}$

## 8. Conclusions and Future Directions

This review has resulted in a mathematically-driven synthesis of deep learning in cardiology based on our incorporation of 79 primary studies together with a theoretical analysis of data representation, optimization dynamics, and principles of trustworthy artificial intelligence. As opposed to conventional reviews that focus on the prediction accuracy of a considered technique, our review examines the mathematical principles needed for clinical implementation of a given method, such as multimodal representation, optimization, uncertainty, fairness, interpretability, or robustness.

Our analysis shows that while the performance of deep learning models in cardiovascular applications is impressive in terms of diagnostic accuracy, many theoretical limitations exist between empirical accuracy and the clinical reliability of techniques under consideration. As can be seen from our literature review, very little attention has been paid to the calibration, uncertainty, fairness, robustness, and correct mathematical representations of different types of cardiac data.

### 8.1. Strategic Directions for Future Research

Future research should focus on four major directions:

1. **Learning based on physiology:** Optimization goals for future AI models for cardiovascular diseases should be adapted to include specific physiological constraints. Physics-informed and knowledge-based learning techniques can potentially increase the reliability of such models by generating biologically plausible solutions without loss of predictive ability.
2. **Consistent assessment of trustworthiness:** Evaluation of AI models in clinical practice should be expanded beyond simple accuracy assessments to include measures of calibration, uncertainty, fairness, interpretability, and robustness. The creation of consistent auditing protocols will allow for more reliable comparison of new models. Such a protocol could also help to create an objective map of attention to trustworthiness for different architectures.
3. **Scalable privacy-preserving learning:** Federated learning and foundation models can be seen as promising approaches to solving the multi-institutional data challenge. In the future, however, mathematically sound approaches should be developed in order to deal with issues around dependent and varied clinical data, efficient communications, and stable convergence. Algorithm 4 outlines a federated training procedure along these lines.
4. **Real-time uncertainty-aware deployment:** In clinical practice, models need to be able to detect unreliable predictions. The use of uncertainty quantification approaches such as Bayesian and ensemble-based techniques can be useful for this purpose.

**Algorithm 4** Privacy-PreservingFederated Trustworthy Cardiac AI Training**Require:** 
Hospitals $\left\{D_{1},D_{2},\ldots ,D_{K}\right\}$, initial model parameters $\theta_{0}$, communication rounds *R***Ensure:** 
Global trustworthy model $\theta^{*}$  1:**for** r=1 to *R* **do**  2:      Server distributes global model $\theta_{r}$ to all hospitals  3:      **for** each hospital k=1,…,K in parallel **do**  4:         Train local model using private cardiac data:$$\theta_{r}^{k}=\theta_{r}-\eta \nabla_{\theta }L_{total}^{k}$$
       where$$L_{total}^{k}=L_{task}+\lambda_{f}L_{fair}+\lambda_{r}L_{robust}$$  5:         Add privacy-preserving noise:$${\tilde{\theta }}_{r}^{k}=\theta_{r}^{k}+N\left(0,\sigma^{2}\right)$$  6:      **end for**  7:      Aggregate local updates:$$\theta_{r+1}=\sum_{k=1}^{K}\frac{n_{k}}{N}{\tilde{\theta }}_{r}^{k}$$  8:      Evaluate global model: calibration, fairness, robustness  9:**end for**10:**return** $\theta^{*}=\theta_{R+1}$

### 8.2. Limitations and Threats to Validity

Despite the systematic and mathematical nature of this review, there are certain limitations to note. First, the analysis was limited to English-language publications only, meaning that it is subject to language bias. Second, the dynamic nature of cardiovascular artificial intelligence means that new strategies may have been developed in the second half of 2025 or 2026, and would not have been included within the timeframe of our literature review. Although this constrains the timeframe to exclude the most recent architectural innovations, it guarantees a solid and well-analyzed body of work for the mathematical model under consideration. Nonetheless, further reviews will be needed in the future. Third, although certain quantitative criteria are used in this review, there is no way to avoid reliance on expert judgment when assessing the mathematical soundness and methodological quality of a publication. Fourth, publication bias may impact the observed trends, since studies demonstrating unsuccessful results tend to be published less frequently. Although our systematic analysis suggests a number of quantitative trustworthiness criteria (RD, TDI, RS), systematic calculation of these criteria for all studies was beyond the scope of the paper, and should be considered one of the main directions for future work. Future studies should focus on validating the suggested mathematical approach through clinical assessments and benchmarking studies.

**Limitations of the Numerical Application of RD and TDI.** The suggested representation deficit (RD) and trustworthiness deficit index (TDI) metrics were not systematically calculated on the basis of the 79 studies covered in this review. The reason for this lies in the diversity of the studies covered in this review and the lack of complete and consistent information necessary for the reproducible calculation of the RD and TDI. Calculating the RD requires comparable data representations and the definition of a mapping procedure and divergence estimation approach. The same applies to TDI calculation, which needs operationally defined metrics, justifiable thresholds, and an adequate evidence base in order to provide valid calibration, fairness, robustness, and explainability results. These requirements were not fulfilled by evidence available from this review. Therefore, we use an illustrative numerical example in Section 7.4; this example is only a demonstration, and does not constitute an analysis of any of the 79 reviewed studies. Systematic calculation of RD and TDI is future work that requires standardized data extraction and calculation procedures.

## Figures and Tables

**Table 1:** Comparison with recent reviews of AI/deep learning in cardiovascular diagnosis.

Prior Review	Scope	Math Formalization Depth	Trustworthiness Dimensions Covered	Gap Filled By This Paper
[20]	PRISMA review and meta-analysis of 85 DL-ECG studies for CVD classification	Empirical/statistical (meta-analysis of diagnostic accuracy); no unified mathematical framework of representation or optimization	Reports prevalence of encryption (4.7%), uncertainty (0%), and explainability (9.4%) as empirical findings	Provides the mathematical/theoretical formalism (representation, optimization, trustworthiness constraints) absent from this empirically focused review
[6]	PRISMA review of 102 AI-ECG studies across disease types, architectures, and methodological innovations	Descriptive/comparative; architecture and performance tables, no formal mathematical synthesis	Explainability, dataset bias/fairness, wearable-related privacy concerns, regulatory frameworks	Offers a unified mathematical treatment of trustworthiness (calibration, fairness, robustness, privacy) rather than a narrative/comparative synthesis
[21]	Systematic review of explainable AI, specifically in cardiovascular imaging	Method-specific (Grad-CAM and related attribution techniques); not a general mathematical framework	Explainability only; does not address calibration, fairness, robustness, or privacy	Extends beyond explainability alone, and beyond imaging to a cross-modality (ECG, imaging, EHR) mathematical trustworthiness framework

**Table 2:** Database-specific search strategies used for the systematic literature review.

Digital Library	Search String
IEEE Xplore	(“deep learning” OR “artificial intelligence” OR “machine learning”) AND (cardiology OR cardiovascular OR cardiac OR ECG OR electrocardiogram) AND (trustworthy OR explainable OR explainability OR fairness OR robustness OR uncertainty OR privacy)
Taylor & Francis	TS=((“deep learning” OR “artificial intelligence” OR “machine learning”) AND (cardiology OR cardiovascular OR cardiac OR ECG) AND (trustworthy OR explainable OR fairness OR robustness OR uncertainty OR privacy))
PubMed	((“Deep Learning”[Title/Abstract] OR “Artificial Intelligence”[Title/Abstract] OR “Machine Learning”[Title/Abstract]) AND (“Cardiology”[Title/Abstract] OR cardiovascular[Title/Abstract] OR cardiac[Title/Abstract] OR ECG[Title/Abstract]) AND (trustworthy[Title/Abstract] OR explainable[Title/Abstract] OR fairness[Title/Abstract] OR robustness[Title/Abstract] OR uncertainty[Title/Abstract] OR privacy[Title/Abstract]))
ScienceDirect	(“deep learning” OR “artificial intelligence”) AND (cardiology OR cardiovascular OR cardiac OR ECG) AND (trustworthy OR explainable OR fairness OR robustness OR uncertainty OR privacy)
Springer	(“deep learning” OR “artificial intelligence”) AND (cardiology OR cardiovascular OR cardiac) AND (trustworthy OR explainable OR fairness OR robustness OR uncertainty OR privacy)
Wiley Online Library	(“deep learning” OR “artificial intelligence”) AND (cardiology OR cardiovascular OR cardiac) AND (trustworthy OR explainable OR fairness OR robustness OR uncertainty OR privacy)
ACM Digital Library	(“deep learning” OR “artificial intelligence”) AND (cardiology OR cardiovascular OR ECG) AND (trustworthy OR explainable OR fairness OR robustness OR uncertainty OR privacy)

**Table 3:** Database-wise study selection summary.

Database	Records Screened (Post-Duplicate)	Abstracts Assessed	Full-Text Assessed
IEEE Xplore	92	46	36
ScienceDirect	49	22	20
ACM Digital Library	30	15	8
SpringerLink	21	7	3
Wiley Online Library	30	27	8
PubMed	19	13	3
Taylor & Francis Online	20	13	1
**Total**	**261**	**143**	**79**

**Table 4:** Relevanceassessment of the 79 included studies across the four research questions.

Sr. No.	Ref.	RQ1			RQ2			RQ3			RQ4			Total
		Relevant	Partial	Irrelevant	Relevant	Partial	Irrelevant	Relevant	Partial	Irrelevant	Relevant	Partial	Irrelevant	Score
1	[29]	✓			✓				✓		✓			3.5
2	[30]	✓					✓	✓			✓			3
3	[31]	✓			✓			✓				✓		3.5
4	[32]	✓				✓		✓					✓	2.5
5	[33]		✓				✓	✓					✓	1.5
6	[34]	✓			✓			✓				✓		3.5
7	[35]		✓			✓			✓		✓			2.5
8	[36]	✓			✓					✓		✓		2.5
9	[37]		✓		✓			✓					✓	2.5
10	[38]		✓			✓			✓			✓		2
11	[39]	✓			✓			✓				✓		3.5
12	[40]		✓		✓			✓			✓			3.5
13	[41]	✓			✓			✓				✓		3.5
14	[42]			✓	✓			✓					✓	2
15	[43]	✓			✓			✓					✓	3
16	[44]	✓			✓			✓			✓			4
17	[45]	✓			✓			✓				✓		3.5
18	[46]	✓			✓			✓			✓			4
19	[47]			✓	✓			✓			✓			3
20	[48]		✓		✓			✓				✓		3
21	[49]	✓			✓			✓			✓			4
22	[50]			✓	✓			✓					✓	2
23	[51]	✓			✓				✓			✓		3
24	[52]		✓		✓					✓		✓		2
25	[53]			✓	✓			✓			✓			3
26	[54]	✓			✓			✓					✓	3
27	[55]		✓			✓		✓					✓	2
28	[56]	✓			✓					✓			✓	2
29	[57]		✓		✓			✓				✓		3
30	[58]			✓	✓			✓			✓			3
31	[59]		✓		✓			✓				✓		3
32	[60]		✓		✓				✓			✓		2.5
33	[61]			✓	✓			✓					✓	2
34	[62]		✓		✓			✓			✓			3.5
35	[63]			✓	✓			✓					✓	2
36	[64]		✓		✓			✓				✓		3
37	[65]	✓					✓	✓				✓		2.5
38	[66]			✓		✓		✓					✓	1.5
39	[67]	✓			✓			✓			✓			4
40	[68]	✓					✓	✓					✓	2
41	[69]	✓				✓		✓			✓			3.5
42	[70]	✓			✓			✓					✓	3
43	[71]			✓		✓		✓				✓		2
44	[72]		✓				✓	✓			✓			2.5
45	[73]	✓			✓			✓					✓	3
46	[74]		✓		✓			✓			✓			3.5
47	[75]	✓				✓		✓					✓	2.5
48	[76]			✓	✓			✓			✓			3
49	[77]			✓		✓		✓					✓	1.5
50	[78]	✓			✓			✓			✓			4
51	[79]		✓		✓				✓				✓	2
52	[80]	✓					✓	✓				✓		2.5
53	[81]	✓				✓				✓		✓		2
54	[82]	✓				✓		✓				✓		3
55	[83]	✓			✓				✓			✓		3
56	[84]			✓	✓				✓				✓	1.5
57	[85]	✓			✓				✓				✓	2.5
58	[86]	✓					✓	✓					✓	2
59	[87]	✓				✓		✓					✓	2.5
60	[88]	✓			✓			✓			✓			4
61	[89]		✓				✓	✓					✓	1.5
62	[90]	✓			✓				✓		✓			3.5
63	[91]	✓					✓	✓				✓		2.5
64	[92]	✓			✓					✓		✓		2.5
65	[93]	✓			✓			✓					✓	3
66	[94]	✓				✓				✓		✓		2
67	[95]	✓					✓	✓				✓		2.5
68	[96]	✓					✓	✓					✓	2
69	[97]	✓					✓	✓					✓	2
70	[98]	✓					✓	✓					✓	2
71	[99]	✓					✓	✓			✓			3
72	[100]	✓					✓	✓			✓			3
73	[101]	✓					✓	✓			✓			3
74	[102]	✓					✓	✓					✓	2
75	[103]	✓					✓	✓				✓		2.5
76	[104]	✓					✓	✓				✓		2.5
77	[105]	✓					✓	✓					✓	2
78	[106]	✓					✓	✓					✓	2
79	[107]	✓					✓	✓					✓	2

Scoring: Relevant = 1, Partial Relevance = 0.5, Irrelevant = 0. The total score is the sum of the four research question ratings (range: 0–4). Each study has exactly one relevance category per research question.

**Table 5:** Qualitative synthesis of evidence characteristics in the included cardiovascular deep learning studies.

Characteristic	Evidence Examined	Synthesis Approach
Clinical application	Diagnosis, prognosis, risk prediction, monitoring, and clinical decision support	Studies were considered according to their cardiovascular condition and intended clinical task.
Data modality	ECG, echocardiography, cardiac MRI, CT, other imaging, clinical records, and multimodal data	The input data modalities reported in the included studies were considered.
Dataset and cohort	Public and institutional datasets, cohort characteristics, and multi-center or external data sources	Dataset sources and cohort descriptions were considered where reported.
Validation strategy	Random data splits, cross-validation, temporal validation, external validation, and prospective evaluation	The reported validation protocols were examined to characterize how model performance was assessed.
Explainability	Feature attribution and other interpretation methods	The review discusses the use of explainability methods and the limited attention given to attribution stability.
Calibration and uncertainty	Probability calibration and uncertainty estimation	The qualitative synthesis identifies limited attention to calibration and uncertainty quantification.
Fairness	Evaluation of performance across demographic or clinical subgroups	The review identifies fairness as an insufficiently addressed dimension in cardiovascular deep learning research.
Robustness	Robustness testing and adversarial evaluation	The review identifies limited evaluation of robustness and adversarial resilience.
Privacy	Methods for privacy-preserving model development or data analysis	The review discusses the limited adoption of privacy-preserving approaches.

Note: The review covers 79 studies. This table provides a qualitative synthesis of the areas of evidence that have been considered, but does not provide the frequency or percentage for each study. The qualitative synthesis of the trustworthiness dimensions is based on the narrative synthesis of the review.

**Table 6:** Qualitative summary of trustworthiness dimensions and their coverage in the reviewed literature.

Trustworthiness Dimension	Reported Level of Attention in Reviewed Literature	Basis
Explainability	Commonly addressed via post-hoc methods (SHAP, LIME, Integrated Gradients), though attribution stability for correlated features is rarely examined	Section 4.1
Calibration	Limited attention; rarely reported alongside predictive performance	Section 1.2
Uncertainty quantification	Rarely addressed; most models produce deterministic outputs without confidence estimation	Section 5.4
Algorithmic fairness	Rarely evaluated explicitly; demographic bias remains largely unexamined	Section 4.2
Adversarial robustness	Rarely evaluated formally; few studies test models under adversarial or perturbed conditions	Section 4.3
Privacy preservation	Limited adoption, constrained by trade-offs with computational efficiency and predictive performance	Section 4.4

## Data Availability

No new data were created in this study. The included studies and their relevance ratings are reported in Table 4.
